# HES1 inhibition overcomes CDK4/6 inhibitor resistance by targeting cancer stemness in lung adenocarcinoma

**DOI:** 10.1186/s13046-026-03771-x

**Published:** 2026-06-27

**Authors:** Alvin Ho Kwan Cheung, Kit Yee Wong, Gordon Yuan-Ho Li, Fuda Xie, Qiuqiu Wu, Thomas Tin Ho Yuen, Pak Lam Hui, Pingmei Huang, Xiaoli Liu, Bonan Chen, Fenfen Ji, Yujuan Dong, Alfred Sze-Lok Cheng, Calvin Sze Hang Ng, Xiang Zhang, Chi Chun Wong, Jun Yu, Wei Kang, Ka-Fai To

**Affiliations:** 1 Department of Anatomical and Cellular Pathology, State Key Laboratory of Translational Oncology, Prince of Wales Hospital, The Chinese University of Hong Kong, Shatin, Hong Kong, China; 2 Department of Medicine and Therapeutics, Institute of Digestive Disease, State Key Laboratory of Digestive Disease, Li Ka Shing Institute of Health Sciences, The Chinese University of Hong Kong, Hong Kong SAR, China; 3 School of Biomedical Sciences, The Chinese University of Hong Kong, Hong Kong SAR, China; 4 Department of Surgery, Prince of Wales Hospital, The Chinese University of Hong Kong, Shatin, Hong Kong, China

**Keywords:** HES1, CDK4/6 inhibitor, Lung adenocarcinoma, SOX9

## Abstract

**Background:**

CDK4 alterations are common in lung adenocarcinoma, but recent clinical trials only demonstrated modest therapeutic responses to CDK4/6 inhibitors. The mechanism of CDK4/6 inhibitor resistance has not been fully characterized.

**Methods:**

Patient-derived organoids and cell lines were tested for their sensitivity to CDK4/6 inhibitors. The drug resistance mechanism relating to stemness pathway was tested by in-vitro and in-vivo assays. Screening of small molecule library was performed to explore potential therapeutic agents that could potentiate the efficacy of CDK4/6 inhibitors.

**Results:**

CDK4/6 inhibitors could inhibit the growth of lung adenocarcinoma patient-derived organoids and cell lines, and its drug resistance was associated with HES1 overexpression. We identified the vital role of HES1 in promoting cancer cell stemness through SOX9 upregulation via the phosphorylation of transcription factor STAT3. Inhibition of HES1 impaired lung cancer spheroid formation, reduced stemness marker expression, and enhanced the sensitivity of the spheroids towards the CDK4/6 inhibitor palbociclib. Knockdown of SOX9 recapitulated the functional effect of HES1 inhibition, confirming its role as a downstream effector mediating the stemness properties of lung cancer cells. Screening of small molecules revealed VR23 as a potent HES1 inhibitor, and it could suppress lung cancer growth and patient-derived organoids in a synergistic manner with palbociclib in-vitro and in-vivo. Moreover, the pSTAT3 inhibitor napabucasin could also afford synergy with palbociclib as well, further confirming the therapeutic vulnerability conferred by the HES1-pSTAT3-SOX9 pathway in potentiating CDK4/6 inhibitors.

**Conclusions:**

Our findings revealed a signalling pathway in which lung adenocarcinoma regulates stemness and tumourigenesis through HES1, and the targeting of this pathway by VR23 or napabucasin supports further preclinical development for CDK4/6 inhibitor combination therapy.

**Supplementary Information:**

The online version contains supplementary material available at https://doi.org/10.1186/s13046-026-03771-x.

## Introduction

Lung adenocarcinoma accounts for significant cancer mortality and morbidity worldwide. CDK4/6 inhibitors have been described as promising therapeutic agents, especially for breast cancer [1]. CDK4/6 inhibitors bind to the ATP pocket of both CDK4 and CDK6 proteins, blocking the function of the cyclin-CDK4/6 complex, thereby inducing G1 phase cell cycle arrest [2]. Emerging evidence indicates that CDK4/6 inhibitors could have non-canonical effects outside of regulating the cell cycle, such as promoting cell senescence, enhancing immunogenicity, and disrupting cancer metabolism [3, 4]. The cell cycle is an important target because multiple oncogenic pathways converge on this cellular process, which is a hallmark of cancer [5]. The recent development of selective CDK4 inhibitors represents the intense interest in this therapeutic pathway [6].

Preclinical findings suggested CDK4/6 inhibitors are promising in the treatment of lung cancer. Palbociclib could overcome acquired resistance to third‐generation EGFR tyrosine kinase inhibitor osimertinib in adenocarcinoma [7]. Another study demonstrated abemaciclib, another CDK4/6 inhibitor, may enhance the radio-sensitivity of lung cancer by inhibiting Rb phosphorylation and causing nutrient stress by reducing intracellular amino acid pools in cells which are subjected to radiotherapy [8]. Despite positive findings in cell line models, the therapeutic response of lung adenocarcinoma patients to CDK4/6 inhibitors appeared modest in clinical trials [9], necessitating further investigation into the molecular mechanisms underlying their efficacy and resistance.

One potential resistance mechanism is related to cancer stemness properties. Cancer stem cells are thought to confer therapeutic resistance due to their slow-dividing cell cycle, enhanced drug efflux mechanisms, and active DNA damage repair [10, 11]. Cancer stemness is tightly regulated by the Notch signaling pathway [12, 13], in which one of its transcriptional regulators is HES1. HES1 belongs to the Hairy and Enhancer of Split family of proteins, acting as an effector related to development and cell fate programming [14]. HES1 has been reported to promote self-renewal in cancer stem cells through modulation of stemness genes and alteration of gene expression in differentiation and proliferative pathways related to therapeutic resistance [15], such as the up-regulation of CD133, ABCG2, Nanog, ALDH1, CD44, and Bmi-1 [16]. Furthermore, HES1 can maintain a population of cancer stem cells during periods of treatment response when differentiation and apoptosis are inhibited [17], such as in colon cancer [18]. However, the function of HES1 in lung adenocarcinoma remains incompletely understood.

In this study, we first examined the therapeutic mechanisms of CDK4/6 inhibitors in lung adenocarcinoma and identified HES1 overexpression as having a key role in mediating resistance to these agents. We dissected the functional role of HES1 and its downstream signaling molecules, as well as the cancer stemness phenotype associated with it. We further explored the therapeutic vulnerability conferred by the HES1 signaling pathway and demonstrated that it holds translational potential as a combination therapy with CDK4/6 inhibitors.

## Methods

### Cell and organoid culture

Lung adenocarcinoma cell lines A-549 (RRID: CVCL_0023), NCI-H23 (RRID: CVCL_1547), NCI-H358 (RRID: CVCL_1559), NCI-H1792 (RRID: CVCL_1495), NCI-H2030 (RRID: CVCL_1517), NCI-H1563 (RRID: CVCL_1475), NCI-H1975 (RRID: CVCL_1511), NCI-H3255 (RRID: CVCL_6831), NCI-H1650 (RRID: CVCL_1483) were obtained from American Type Cell Culture (ATCC). All human cell lines have been authenticated using STR profiling. Each cell line was maintained as per instructions by the ATCC. Patient-derived organoids were produced by dissecting the tumour samples and digesting the dissected tissue in collagenase-digestion buffer, and resuspending into Matrigel matrix (356231, Corning) for 30 min. Following Matrigel polymerisation, lung cancer specific organoid culture media complemented by growth supplements were added and then incubated at 37 °C, 5% CO2 humidity. All experiments were performed with mycoplasma-free cells. The study was approved by ethics committee of the Chinese University of Hong Kong.

### Immunohistochemistry and clinical samples

Immunohistochemistry (IHC) was carried out on tissue microarray using the Benchmark XT autostainer (Ventana, Tucson, AZ) machine. The microarray was incubated with monoclonal antibodies specific against CDK4 (sc-56277; Santa Cruz Biotechnology, Inc), HES1 (sc-166410; Santa Cruz Biotechnology, Inc.) and SOX9 (#82630; Cell Signaling Technology). Ki67 (#9129, Cell Signaling Technology) and cleaved Caspase 3 (#9661, Cell Signaling Technology) antibodies were used in mice xenografts. UltraView DAB (Ventana, #760–500) was used to detect the primary antibodies. The tissue microarray was generated from a cohort of lung adenocarcinoma patients from the years 1995 to 2010, at the Prince of Wales Hospital, Hong Kong. Tissue samples were formalin-fixed and embedded in paraffin. Assessment of the immunohistochemical findings were performed by pathologists blinded to the nature of the samples.

### Cell transfection and in-vitro functional studies

All transfection assays with siRNAs were performed using Lipofectamine™ 2000 Transfection Reagent (Invitrogen). The pharmacologic agents napabucasin and VR23 were purchased from Selleckchem. All siRNA against CDK4, HES1, and SOX9 were obtained from Qiagen. The entirety of the HES1 and SOX9 backbone was cloned onto a pcDNA3.1(+) backbone. Transfection of the plasmid for the overexpression of HES1 and SOX9 was carried out with the FuGene HD transfection reagent (Promega).

Cell proliferation was assessed using CellTiter 96 Non-Radioactive Cell Proliferation Assay (Promega, Madison, WI). Colony formation assays were performed by seeding the transfected cells in a 6-well plate and incubated for 7–10 days, and colonies counted with 0.2% crystal violet staining. Cell invasion assays (354,480, Corning) were performed as per the manufacturer’s instructions.

In regard to flow cytometry analyses, The FITC Annexin V Apoptosis Detection Kit I (BD, Franklin Lakes, NJ) was used to gate cells undergoing apoptosis. Cell cycle analysis was performed by staining with propidium iodide (Sigma-Aldrich) after fixation of cells in 70% ethanol overnight. The samples were then analysed on the BD LSRFortessa cell analyser platform.

### Quantitative polymerase chain reaction

Total RNA extraction was performed with the E.Z.N.A.® Total RNA Kit I (R6834-02, Omega Bio-tek). RNA concentration was measured by NanoDrop 2000 Spectrophotometer (Thermofisher). Following RNA extraction, reverse transcription was performed using PrimeScript RT reagent kit (Takara). Real time qPCR was performed using the Quantstudio Flex 6 real time PCR system (ThermoFisher), with the TB Green Premix Ex Taq kit (Takara).

### Protein extraction, Western blot analysis

Protein lysate was obtained by cell lysis in the RIPA buffer and then separated by SDS-PAGE and then transferred onto PVDF membranes (GE Healthcare, Piscataway, NJ). The primary antibodies specific against Phospho-MEK1/2 (#9121), MEK (#9122), p21 (#2946), p27 (#3688), Phospho-Rb (Ser807/811) (#9308), cyclin D1 (#2978), cleaved PARP (Asp214) (#9541), PARP (#9542), STAT3 (#9139), pSTAT3 (#9145), Sox9 (#82,630), Nanog (#4903), activated β-catenin (#8814), Ubiquitin (#58935S), HSF1 (#4356), BiP (#3177 T), eIF2a (#5324 T), p-eIF2a (#3398 T), HA-tag (#3724S), CHOP (#2895 T) were obtained from Cell Signaling Technology (CST; Danvers, MA). Antibodies against CDK4 (sc-56277) and HES1 (sc-166410) were obtained from Santa Cruz Biotechnology. Antibodies targeting HSP72 (#BD-PN0205), HSP90a (#BD-PM3480) were purchased from BioDragon. The p-HSF1 (#AB76076) were purchased from Abcam, and the HSP70 antibody (#MA3-007) was purchased from Thermo Fisher Scientific. GAPDH (#2118; CST) expression was used as loading control. The membranes were then incubated with primary antibodies on a rocking incubator at 4 °C overnight, washed three times with 5% BSA in TBST, and then incubated with HRP-conjugated secondary antibodies at room temperature for 1 h. Anti–mouse IgG-HRP (Dako P0260, 1:3,000) and anti–rabbit IgG-HRP (Dako P0448, 1:2,500) were used as secondary antibodies. The membranes were developed and captured on the ChemiDoc XRS machine (Bio-Rad) using ImageLab software.

### Fluorescence in situ hybridization (FISH)

FISH analyses are performed on tissue microarrays and paraffin-embedded formalin-fixed samples. CDK4 probe (FG0033, Abnova) is used to detect the copy number changes with CEN12 as a control. PreTreatment Kit 1 (KA2375, Abnova) is used for the pretreatment of paraffin-embedded formalin-fixed tissue sections.

### Immunoprecipitations and ChIP-qPCR

ChIP was performed with the SimpleChIP Enzymatic Chromatin IP Kit (Magnetic Beads; #9003) as per manufacturer's (CST) protocol with some modifications. Following chromatin digestion, the nuclei was pelleted as per manufacturer’s instructions. The nuclear envelope was then broken down by snap freezing, immersing the nuclear pellet in liquid nitrogen three times. Following cell culture and fixation of samples, cross-linked chromatin was sheared by the Covaris sonication system (S220) to desired fragment lengths of 300–500 base pairs. The sonicated chromatin were immunoprecipitated by incubation with Stat3 mouse mAb (#9139), pStat3 rabbit mAb (#9145). ChIP-qPCR was performed using primers binding to the putative binding sites of the Sox9 promoter, with the sequence 5’-TATGGATTATTACGGAGGAACAGCG and 5'-CTCCGACATTTCTTTTCACTGCTCT. qPCR was performed as described above, with subsequent calculation of the relative enrichment of amplicons between the target antibodies and the anti-IgG antibody.

### Luciferase activity assays

The promoter of SOX9 was sub-cloned into the pGL3-Basic vector (Promega). Deletion was made at the putative binding site of STAT3 (Deletion 2: ATTTCTAAGCACT deletion), and at a site that were few base pairs upstream to the putative binding site of STAT3 (Deletion 1: ATTTGCTTCAAAAGA deletion). The luciferase reporter activity was subsequently measured with the Dual-Luciferase Reporter Assay System (Promega) according to the assay protocol.

### Bioinformatics analysis

The cancer genome atlas (TCGA) clinical data for lung adenocarcinoma patients in this study was analyzed on the cBioportal database (https://cbioportal.org). Gene set enrichment analysis was performed using gene sets provided by the MSigDB database (https://www.gsea-msigdb.org/gsea/msigdb). For the functional enrichment analysis regarding the HES1 expression level, the grouping of patients were designated as follows. The patients whose tumours harbored the lowest 10% of the overall HES1 expression were designated as HES1^low^, and those who harbored the highest 10% of the HES1 expression were designated as HES1^high^. The gene set enrichment analysis and all comparisons for gene expression alterations were performed by comparing these HES1^high^ and HES1^low^ patients. Gene ontology analysis was carried out to identify enriched gene set using the R package ‘clusterprofiler’ (version 3.18). For single cell sequencing analysis, further analysis was performed on the lung adenocarcinoma single cell sequencing dataset GSE131907 on publicly available GEO repository. The R package ‘Seurat’ (version 4.0.2) was used to perform dimension reduction with t-distributed stochastic neighbor embedding analysis, cell clustering, and visualization.

### Animal studies and xenograft models

A549 cancer cells (1 × 10^6^ cells/mouse, diluted in 0.1 ml PBS) were subcutaneously injected into the dorsal flank of Balb/c NOD-SCID mice, with the cells having been transduced with lentiviral vectors carrying shControl, shHES1-1, and shHES1-2 ((Vector backbone: pLKO.1; target sequence: 5’-GGACAUUCUGGAAAUGACA-3’(shHES1-1); 5’- GCCAGCUGAAAACACUGAUU-3’ (shHES1-2)). The mice were sacrificed 30 days after inoculation, and the xenografts were harvested. Administration of Palbociclib was by the intraperitoneal route, at a dosage of 20 mg/kg every 3 days. Administration of VR23 was by the intraperitoneal route, at a dosage of 40 mg/kg every 2 days. In vivo limiting dilution assay was performed by subcutaneous injection of siHES1-transfected A549 cancer cells at 5 different doses (1000 cells, 2500 cells, 5000 cells, 10,000 cells, 20,000 cells). Eight tumours were injected for each dose in each group, and tumours were injected to the subcutaneous tissue of left and right flank of each mouse. Number of mice was 20 per group (5 doses × 4 mice each bearing 2 tumours). All animal work was approved by the Animal Experimentation Ethics Committee of the Chinese University of Hong Kong.

### Molecular docking analysis

The online softwares PrankWeb and DeepSite were used to predict the ligand binding sites of HES1 protein based on its three dimensional structure. A compound library consisting of 4,511 molecules with anti-cancer effects was used for the docking experiment. Three dimensional structures of these compounds were downloaded from the PubChem. AutoDock 4.2.6 was used to perform molecular docking in which the binding affinity between the predicted binding sites between the drug and its binding pocket on HES1 was calculated. The Lamarckian genetic algorithm was applied for the docking procedure, with detailed parameters set as follows: energy grid box of 50 × 50 × 50 Å; energy grid spacing of 0.375 Å; a population size of 150 individuals; a maximum number of energy evaluations of 2.5 × 10^6; a maximum number of generations of 2.7 × 10^4; and a gene mutation rate of 0.02. Four independent docking predictions were carried out, and a binding energy of < −12.16 kcal/mol that corresponded to a nanomolar Ki for the ligand-receptor interaction was determined as the screening cutoff. Interactions between the drug and its target were visualized using PyMOL 2.3.

### Drug synergy analysis

Cells were seeded at 0.01 × 10^6^ per well on a 96-well plate. After 24 h, cells were complemented with the drugs palbociclib and napabucasin. To prepare working solutions, the drugs were serial diluted in RPMI/DMEM media. The cells were incubated for another 24 h to allow the drugs to take effect. CCK-8 (#HY-K0301, MCE) was used to measure viability of the cells as per manufacturer’s protocol. Absorbance values were then measured by a plate reader (SpectraMax iD3). The highest single agent (HSA) model was used to determine the degree of synergy between the two drugs. Analyses was conducted through the website SynergyFinder web tool (https://synergyfinder.fimm.fi/). The HSA model was chosen over Bliss independence and Loewe additivity model because of its conservative and stringent threshold applied to synergy and lower vulnerability to an outlier single potent drug, and the latter models were also limited by assumptions that the drug combinations acted on independent pathways, or required dose–response curves that were accountable by sigmoid or logistic functions [19].

### Statistical analyses

Continuous variables were expressed as means ± SD. Comparison between treatment groups was performed using a two-tailed unpaired or paired sample Student's t-test where appropriate. Data was acquired from experiments performed in triplicate unless otherwise specified. *p*-values < 0.05 were considered statistically significant. Survival difference was studied using the log-rank statistics. Analyses were performed with the SPSS statistics software (version 20, SPSS Inc., Chicago, IL) and the Graphpad Prism software (version 8.0.1).

## Results

### CDK4 alterations are common in lung adenocarcinoma

**Fig. 1:**
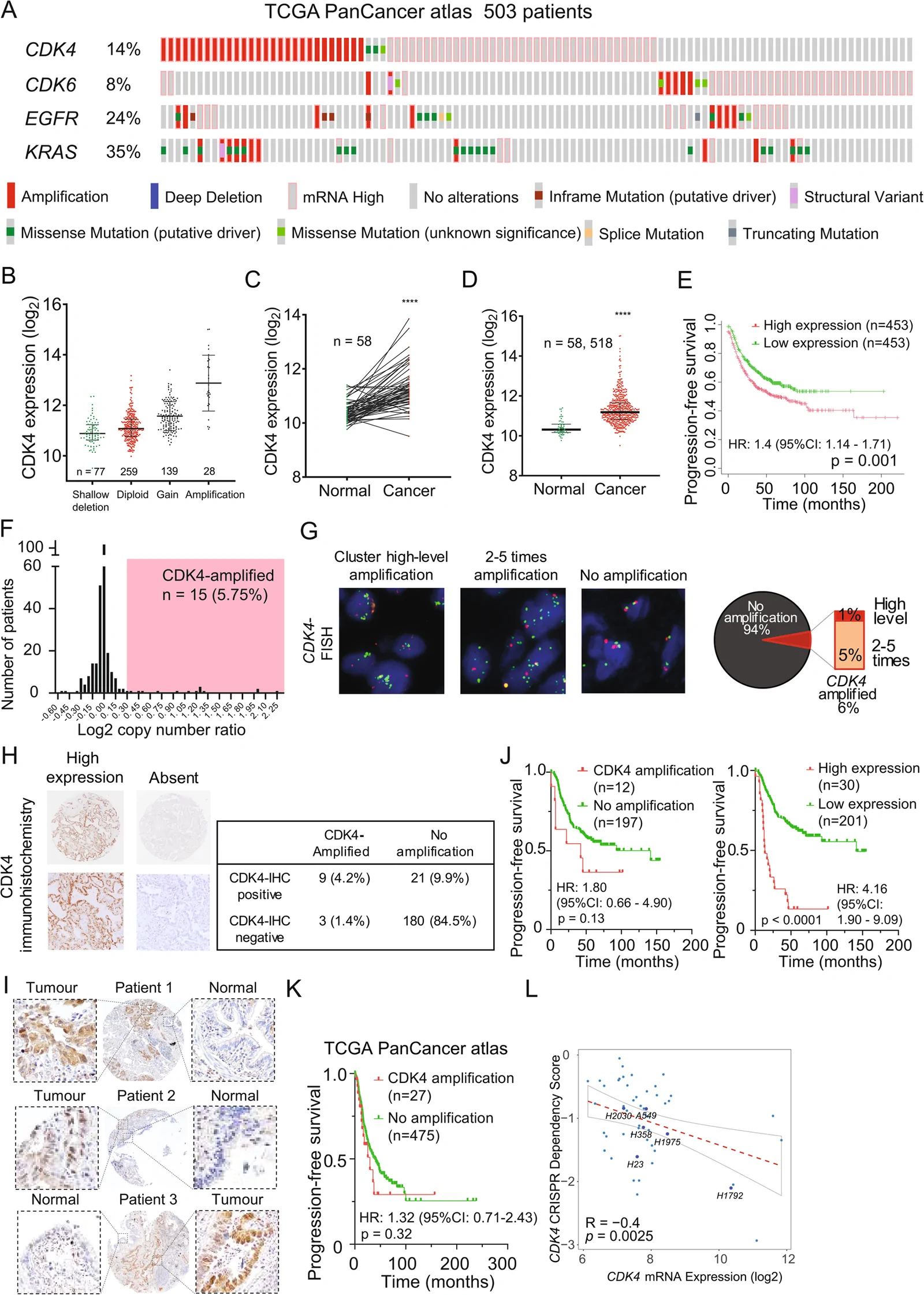
CDK4 alterations are common in lung adenocarcinoma** A** Overview of CDK4, CDK6, EGFR, and KRAS status in 503 patients in the TCGA PanCancer Atlas. **B** *CDK4* RNA expression correlated with copy-number changes. **C**-**D** *CDK4* RNA expression in (**C**) paired (Paired Student's t-test) and (**D**) unpaired cases of adenocarcinoma (Student's t-test). **E** Survival curve modified from the Kaplan–meier plotter database which includes TCGA and GEO lung adenocarcinoma dataset for analyses. **F** 15 patients (5.75%) were positive for CDK4 amplification on sequencing in the in-house patient cohort. **G** Fluorescence in situ hybridization (FISH) analysis in the in-house cohort. Green probe, CDK4. Red probe, CEN12. **H** Immunohistochemical staining of CDK4 on tissue microarray. Table showed the correlation between the immunohistochemistry and FISH results. **I** CDK4 staining on tissue microarray with tumour and normal cellular components. **J** Kaplan–Meier survival curves for progression-free survival in CDK4-amplified and high-expression patients in the in-house lung adenocarcinoma cohort. **K** Kaplan–Meier survival curves for progression-free survival in the TCGA cohort for patients with CDK4-amplified tumours. **L** Dependency on CDK4 correlated to baseline CDK4 expression in lung cancer cell lines (Pearson’s correlation). HR, Hazard ratio. CI, confidence interval. *p < 0.05, **p < 0.01, ***p < 0.001, ****p < 0.0001

CDK4 alterations are frequent in lung adenocarcinoma, and these are mainly represented by gene amplification and overexpression (Fig. 1A). Meanwhile, CDK6 aberrations are much less common, with mostly overexpression and only few amplifications. The CDK4 alterations could co-occur with common driver mutations that were found in the Asian (EGFR) or Caucasian (KRAS) populations, suggesting that CDK4 remained potentially targetable across different driver mutation backgrounds (*p >* 0.05 for mutual exclusivity of alterations).

Among cancers, CDK4 expression in lung cancer was high (Supplementary Fig. 1 A). This increase in gene expression could be due to low-level copy number gain or amplification (Fig. 1B). Consistent with this finding, cancer cells were found to have higher expression of CDK4 compared to normal (Fig. 1C-D). From the DepMap database, it was observed that CDK4 copy number level was highly correlated with its mRNA expression in lung cancer cell lines (R = 0.94, *p <* 0.0001, Supplementary Fig. 1B), while the correlation of mRNA expression and protein intensity of CDK4 by mass spectrometry was less prominent (R = 0.6, *p =* 0.002, Supplementary Fig. 1 C).

In published datasets, patients with increased CDK4 expression in their tumours had a worse outcome (Fig. 1E and Supplementary Fig. 1D). Given the frequency of occurrence and the prognostic impact of CDK6 were much less profound in lung adenocarcinoma than CDK4, we continued to investigate the impact of CDK4 alterations on lung carcinogenesis.

Of 261 lung adenocarcinoma patients, we identified 15 patients (5.75%) that were positive for *CDK4* amplification on sequencing (Fig. 1F). Fluorescence in-situ hybridization (FISH) further validated this finding (Fig. 1G). We then examined if there was a correlation between protein expression and gene amplification by performing immunohistochemistry on tissue microarray (Fig. 1H, Supplementary Fig. 2 A). Normal cells did not exhibit CDK4 immunostaining (Fig. 1I, Supplementary Fig. 2B). It was observed that while CDK4 overexpression could occur in the presence (4.2%) or absence (9.9%) of *CDK4* amplification, the adverse prognostic effect of gene overexpression was more profound than the presence of amplification alone (Fig. 1J). Published dataset also confirmed that a worse prognosis was associated with overexpression of *CDK4* (Fig. 1E) but not amplification alone (Fig. 1K). From DepMap, CRISPR dependency data confirmed that the cell line dependency on CDK4 was correlated to its baseline *CDK4* mRNA expression (Fig. 1L), suggesting that CDK4 exhibited essential oncogenic function in lung cancer cells and this was related to its copy number status and mRNA expression.

### CDK4 exerts oncogenic effect in lung cancer and is a potential therapeutic target

**Fig. 2:**
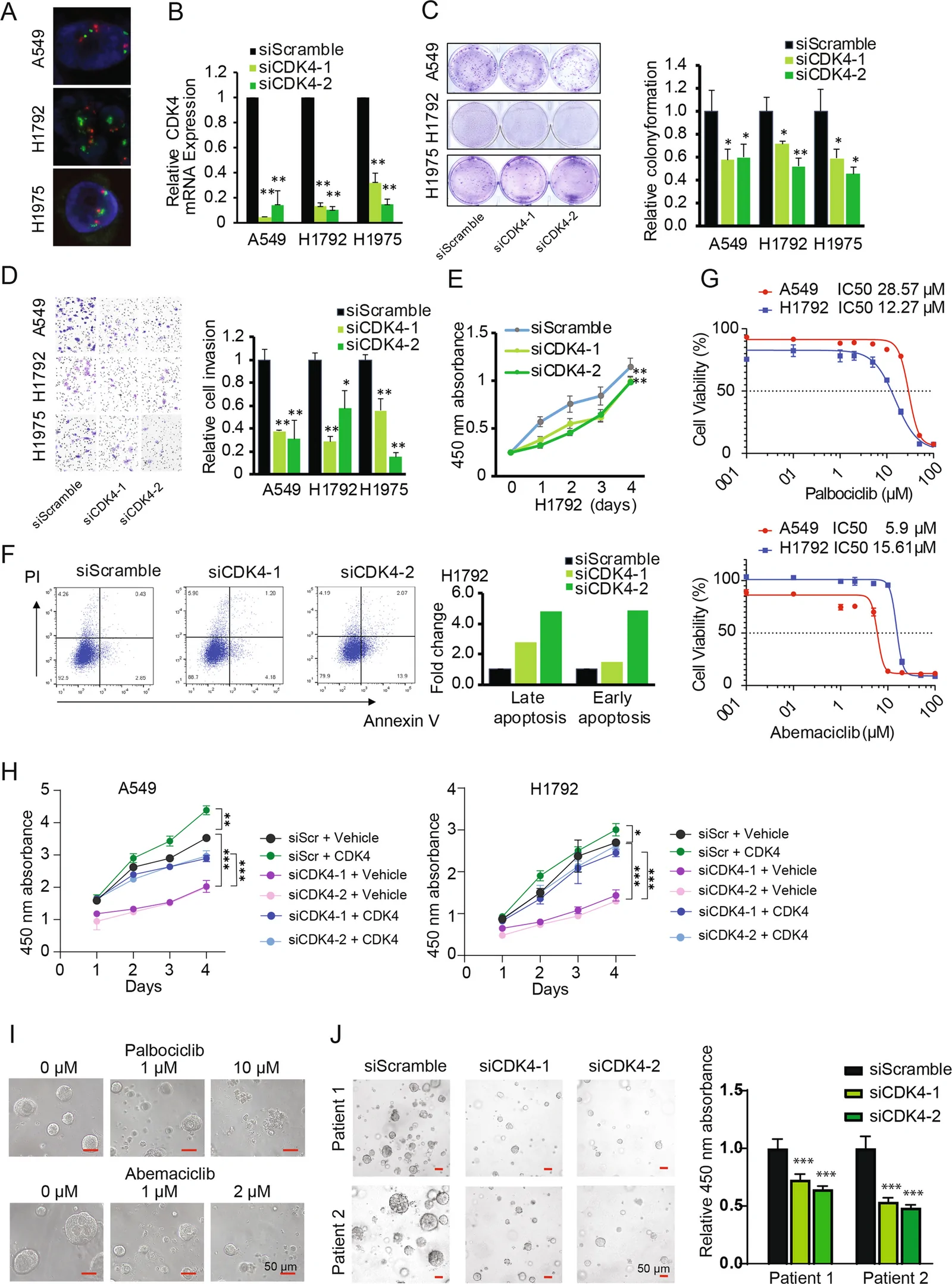
CDK4 exerts oncogenic effect in lung cancer and is a potential therapeutic target. **A** CDK4 fluorescence in situ hybridization (FISH) analysis in cell lines. The cell line H1792 was unique in its presence of CDK4 amplification. Green probe, CDK4. Red probe, CEN12. **B** Knockdown of CDK4 by siRNA in cancer cells. **C**, **D** and **E** (C) colony formation, (D) invasiveness, and (E) proliferation were inhibited upon CDK4 knockdown in cancer cells. Student's t-test. **F** Increased cell apoptosis upon siCDK4 transfection. **G** Dose–response curves of cell lines treated with palbociclib (top) or abemaciclib (bottom). **H** Cell proliferation assay on siCDK4 transfection, with or without re-expression of CDK4 with a siCDK4-resistant plasmid construct. Student's t-test. I Representative images of patient-derived organoids treated with increasing concentrations of palbociclib (0, 1, 10 µM) or abemaciclib (0, 1, 2 µM). Scale bar = 50 µm. **J,** CDK4 knockdown also reduced the organoid size in patient-derived lung cancer organoids. Student's t-test. siScr, siScramble. Scale bar = 50 µm. *p < 0.05, **p < 0.01, ***p < 0.001

To confirm the functional significance of CDK4 overexpression in lung adenocarcinoma, we performed knockdown experiments in vitro. Among the *KRAS*-mutated cell lines A549, H1792, and the *EGFR*-mutated cell line H1975, the cell line H1792 was unique in its presence of high level CDK4 amplification (Fig. 2A). Knockdown of *CDK4* by siRNA (Fig. 2B) showed that the colony forming abilities of the cell lines were consistently decreased (Fig. 2C). The invasion abilities of the cell lines were also decreased (Fig. 2D). The inhibitory effect of *CDK4* knockdown could be accounted for by decreased cell proliferation (Fig. 2E) together with increased cell apoptosis (Fig. 2F). Together, these findings showed that CDK4 plays an important oncogenic role in lung adenocarcinoma.

We further treated the *KRAS*-mutated lung cancer cell lines with CDK4/6 inhibitors. *KRAS* mutated adenocarcinoma was chosen for study because of the relative lack of established targeted therapy for these patients, and the modest tumour response associated with KRAS G12C inhibitors [20]. It was observed that both cell lines exhibited decreased viability when given palbociclib and abemaciclib, in micromolar concentrations (Fig. 2G). The cell proliferation could be partially restored by re-expressing CDK4 with a siRNA-resistant construct in siCDK4 transfectants, confirming that the observed inhibitory effects of siCDK4 was indeed CDK4-specific (Fig. 2H).

Importantly, when patient-derived organoids were treated with CDK4/6 inhibitors, significant decrease in the size of individual organoids was found (Fig. 2I), suggesting that CDK4/6 inhibitors were potentially efficacious in lung adenocarcinoma. To confirm that the effect of CDK4/6 inhibitors was truly brought about by the inhibition of these CDK kinases, we performed knockdown of *CDK4* on patient-derived organoids. *CDK4* knockdown phenocopied the inhibitory effect of CDK4/6 inhibitors on organoids (Fig. 2J). Taken together, these findings demonstrated the oncogenic effect of CDK4 in lung cancer and its potential as a therapeutic target.

### HES1 mediates resistance of CDK4/6 inhibitors in lung adenocarcinoma

**Fig. 3:**
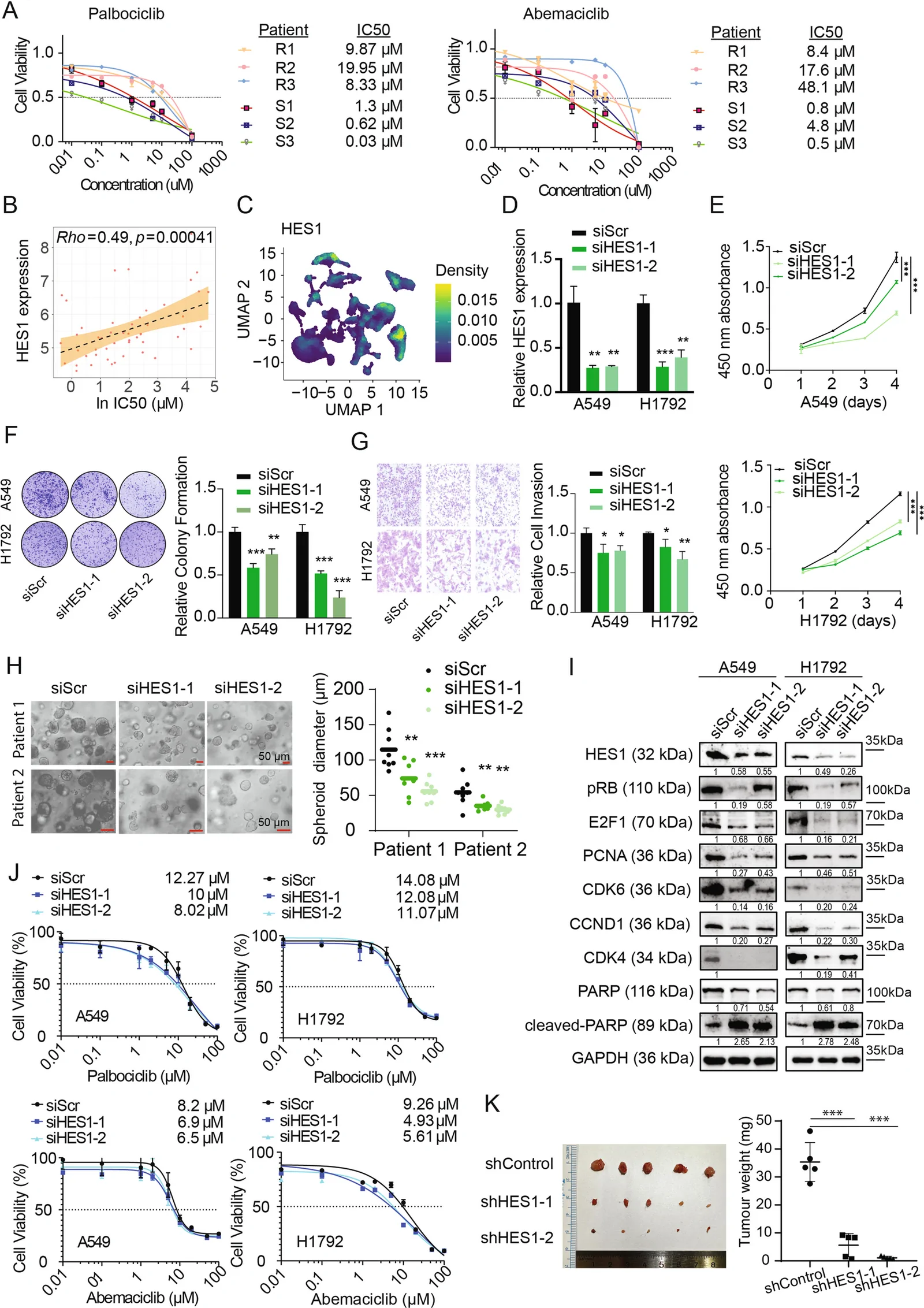
HES1 mediates resistance of CDK4/6 inhibitors in lung adenocarcinoma. **A** Dose–response curves of patient derived organoids treated with palbociclib (left) or abemaciclib (right). Patient with higher drug sensitivity were designated in group S1-S3, while those with lower drug sensitivity were grouped in R1-R3. **B** Spearman correlation between HES1 expression and drug sensitivity in the GDSC dataset. **C** UMAP projection showed HES1 expression distribution in various cell types in the cancer tissue. **D,** HES1 mRNA expression following siRNA-mediated knockdown. **E**,** F**, and** G** HES1 knockdown resulted in reduction in (**E**) cell proliferation, (**F)** Colony formation and (**G**) cell invasion. Student's t-test. **H** In patient-derived organoids, inhibition of organoid growth was observed on siHES1 transfection. Student t-test. **I** Western blot analysis of cell cycle and apoptosis markers after HES1 knockdown. **J** Dose–response curves showed HES1 knockdown sensitizes A549 and H1792 cells to CDK4/6 inhibitors palbociclib and abemaciclib. The IC50 value was given for each group. K, Xenograft formation upon subcutaneous inoculation of shRNA-treated A549 tumour cells in mice. *n =* 5 mice per group. Student's t-test. *p < 0.05, **p < 0.01, ***p < 0.001. siScr, siScramble. Quantification of band intensity was given for Western blot, normalized to the intensity of GAPDH

To unravel potential determinants of CDK4/6 inhibitor efficacy in lung adenocarcinoma, we sought to identify the differential gene expression of patient-derived organoids which exhibited a higher level of CDK4/6 inhibitor resistance on the transcriptome level. We separated three pairs of organoids into CDK4/6 inhibitor sensitive (patients S1-S3) group and less sensitive (patients R1-R3) group, based on their response towards palbociclib and abemaciclib (Fig. 3A). Afterwards, RNA-sequencing was performed on these organoids to obtain a list of genes that were differentially expressed in the less sensitive group.

To narrow down the significant genes that were truly associated with CDK4/6 inhibitor resistance, we used the Genomics of Drug Sensitivity in Cancer (GDSC) database to investigate the response of cancer cell lines towards palbociclib and the relationship with their transcriptome profile. Reassuringly, some cell lines with poorer response towards palbociclib were found to have a higher expression of *CCNE1* and *CCNE2*, in keeping with the findings in clinical trials [21, 22] (Supplementary Fig. 3 A). The overexpression of these genes in resistant cell lines were consistent with the roles of cyclin E1 and cyclin E2 as downstream effectors of CDK4 and as mediators of cell cycle progression [23]. Meanwhile, the mRNA expression of *CDK4*, as well as the *RB* copy number status, and *CDK4* copy number status, did not correlate with drug response (Supplementary Fig. 3A-B).

**Fig. 4:**
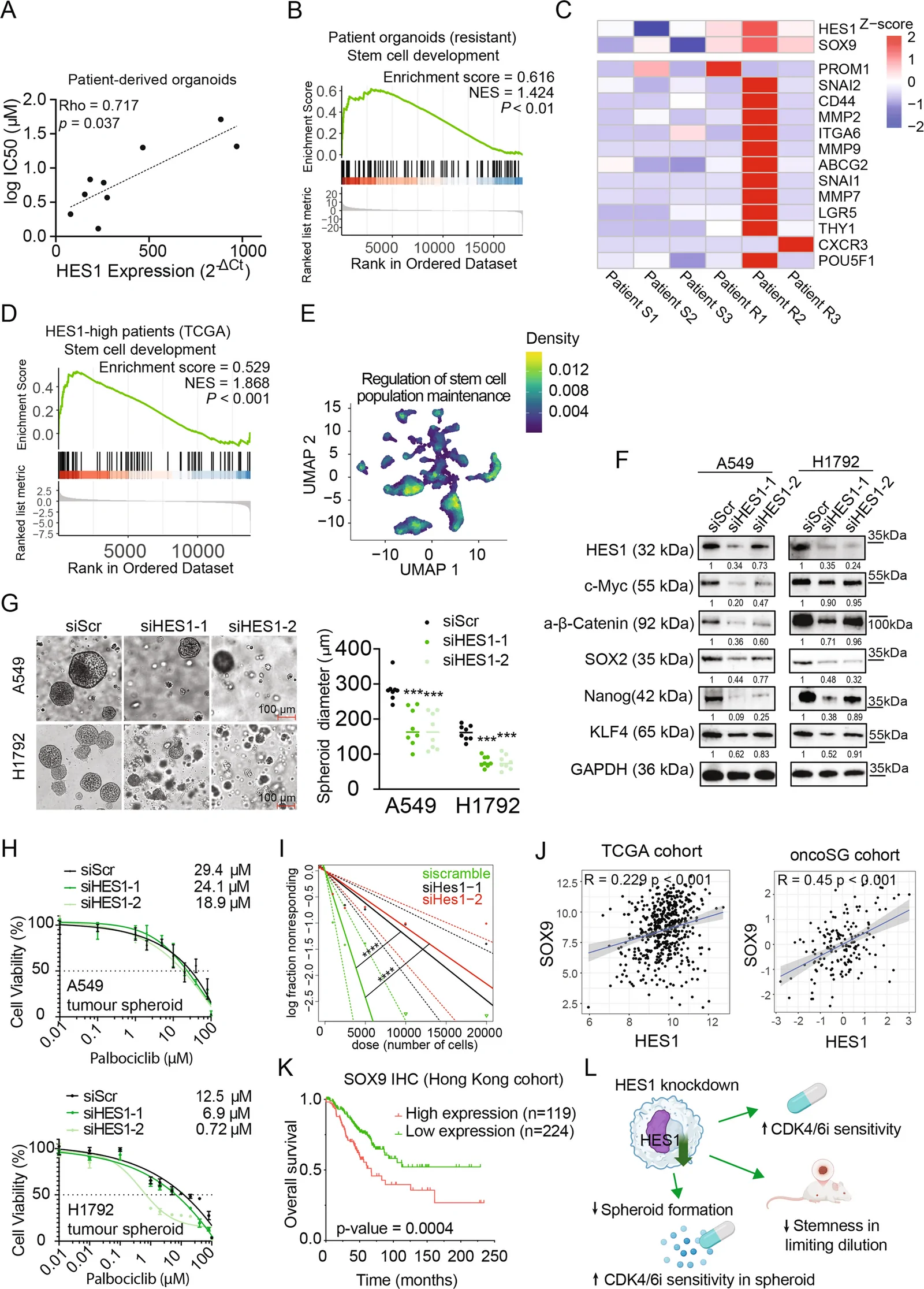
Cancer stemness properties are mediated by HES1 and contributes to drug resistance. **A** Correlation of HES1 mRNA expression as determined by quantitative PCR and the drug response towards Palbociclib in an additional cohort of 9 patient-derived organoids. Spearman correlation. **B** Gene set enrichment analysis (GSEA) of patient derived organoids exhibiting lower drug sensitivity (Patients R1-R3) compared to those exhibiting higher drug sensitivity (Patients S1-S3). **C** Heatmap showing differential gene expression in patient derived organoids exhibiting lower drug sensitivity (Patients R1-R3) compared to those exhibiting higher drug sensitivity (Patients S1-S3). **D** Gene set enrichment analysis (GSEA) of HES1-high lung adenocarcinoma samples in the TCGA cohort. **E** UMAP projection showed the density distribution of stemness-related gene set expression in cancer cells. **F** Western blot analysis demonstrated the effect of HES1 knockdown on the expression of stemness-related proteins in A549 and H1792 cells. a- β-catenin, activated beta-catenin. **G** HES1 knockdown reduced tumor spheroid formation in cell lines. Student t-test. **H** HES1 knockdown resulted in an increased palbociclib sensitivity in tumour spheroids. The IC50 value was given for each group. **I** In vivo limiting dilution assay performed by subcutaneous injection of siHES1-transfected A549 cancer cells at 5 different doses (1000 cells, 2500 cells, 5000 cells, 10,000 cells, 20,000 cells). Eight tumours were injected for each dose in each group. **J** Correlation between HES1 and SOX9 expression in the TCGA cohort and the oncoSG cohort. **K** Immunohistochemistry on tissue microarray and survival analysis in an in-house cohort. High expression was defined by strong or moderate staining, and low expression was defined by weak or absent staining. **L** Schematic diagram summarizing the effect of HES1 knockdown in lung cancer cells. *p < 0.05, **p < 0.01, ***p < 0.001, ****p < 0.0001. siScr, siScramble. Quantification of band intensity was given for Western blot, normalized to the intensity of GAPDH

We examined the candidate genes that were overexpressed in both the resistant patient derived organoids and the resistant cell lines from GDSC. Among all differentially expressed genes, *HES1* stood out due to its significant correlation with drug resistance (Fig. 3B) in lung cancer cell lines, as well as its overexpression in patient-derived organoids that exhibited drug resistance (Fig. 4C). *HES1* RNA level was found to be elevated in non-small cell lung cancer, colorectal cancer and pancreatic cancer [24]. A549 and H1792 had a relatively high HES1 expression among *KRAS*-mutated lung cancer cell lines (Supplementary Fig. 4 A). In lung adenocarcinoma, the overexpression of *HES1* was largely limited to the cancer cells (Fig. 3C, Supplementary Fig. 4B), suggesting that it could be a cancer cell-specific therapeutic target. Mechanistically, HES1 exerted an oncogenic effect on lung cancer cell lines, as *HES1* knockdown (Fig. 3D) resulted in consistent reduction in cell proliferation (Fig. 3E), colony formation (Fig. 3F), and cell invasion (Fig. 3G). In patient-derived organoids, inhibition of organoid growth was observed on siHES1 transfection (Fig. 3H).

Given the correlation between *HES1* overexpression and palbociclib resistance, we reasoned that HES1 may have activated the cell cycle to counteract the effect of CDK4/6 inhibition. Indeed, *HES1* knockdown caused the reduction in expression of molecules involved in cell cycle progression, such as CDK4, CDK6, cyclin D1, and phosphorylated-Rb (Fig. 3I). There was also an increase in the apoptosis marker cleaved-PARP (Fig. 3I). The efficacy of palbociclib and abemaciclib were consistently increased in *HES1*-knockdown cell lines, indicating that HES1 expression at least partially antagonized the drug effect of these two inhibitors (Fig. 3J).

We then confirmed the oncogenic function of HES1 using in-vivo model. Cancer cells were treated with shHES1 and subsequently inoculated subcutaneously into nude mice. Cells with *HES1* knockdown showed significantly smaller xenograft formation than the control group (Fig. 3K). Collectively, these data demonstrated the oncogenic properties of HES1 in lung adenocarcinoma and its inhibition could enhance the efficacy of Palbociclib and Abemaciclib.

### Cancer stemness properties are mediated by HES1 and contributes to drug resistance

To investigate the downstream signalling pathway that contributed to drug resistance, we performed geneset enrichment analyses (GSEA) on the transcriptomic data of the patient derived organoids that exhibited a low CDK4/6 inhibitor sensitivity. Analysis of an additional cohort of 9 patient-derived organoids was also performed, and found a positive correlation between *HES1* mRNA expression and a reduction of drug responsiveness, in keeping with the GDSC findings (Fig. 4A). GSEA desmonstrated that pathways related to cancer stemness, including stem cell development and regulation of stem cell proliferation, were found to be enriched in these resistant organoids (Fig. 4B, Supplementary Fig. 5 A). We explored the genes that were responsible for this enrichment, and found that one or more of the stemness related genes were overexpressed in the resistant organoids (Fig. 4C), compared to the drug sensitive group.

To validate our patient-derived organoid findings, we identified patients with high HES1 expression in the TCGA cohort and performed the same geneset enrichment analyses (Fig. 4D, Supplementary Fig. 5B, C, D). Consistent with the previous findings, the stem cell development pathway was also enriched in the HES1-high TCGA patients (Fig. 4D), confirming that cancer stemness was mediated by HES1 overexpression. In the single cell sequencing data, the increase in stemness-related gene expression was demonstrated in the HES1-overexpressed cancer cell population (Fig. 4E, Supplementary Fig. 5E). In keeping with the informatics finding, a number of stemness related markers were found to be downregulated when cancer cells were subjected to *HES1* knockdown in-vitro (Fig. 4F). Tumour spheroid formation was also inhibited by siHES1 transfection, suggesting a reduction in stemness properties (Fig. 4G). Furthermore, when such spheroids were treated with palbociclib, the efficacy of the drug was increased in siHES1 tumour spheroids, suggesting that the reduction of stemness properties in cancer spheroids was able to potentiate the effect of CDK4/6 inhibition (Fig. 4H). Limiting dilution assay in vivo also demonstrated a reduction in stemness properties when siHES1-transfectants were subcutaneously injected into mice to form xenografts (Fig. 4I).

Within the stemness-related geneset, the SOX family members, including *SOX2*, *SOX4* and *SOX9*, showed overexpression in HES1-high tumours (Supplementary Fig. 5B, C, D), as well as a significant positive correlation with the *HES1* expression (Fig. 4J). We decided to focus on SOX9 in the subsequent analyses, because we found strong positive correlation of *HES1* and *SOX9* expression in the published literature (Fig. 4J, Supplementary Fig. 5D), as well as a worsened survival in patients with high *SOX9* RNA expression (Supplementary Fig. 6 A). In-house analyses using SOX9 immunohistochemistry on tissue microarray also demonstrated a poorer overall survival with high SOX9 protein expression (Fig. 4K, Supplementary Fig. 6B). Taken together, HES1-mediated stemness properties appeared to confer CDK4/6 inhibitor resistance (Fig. 4L), and this may be brought about through the overexpression of SOX9.

### HES1 mediates SOX9 expression through STAT3 phosphorylation

**Fig. 5:**
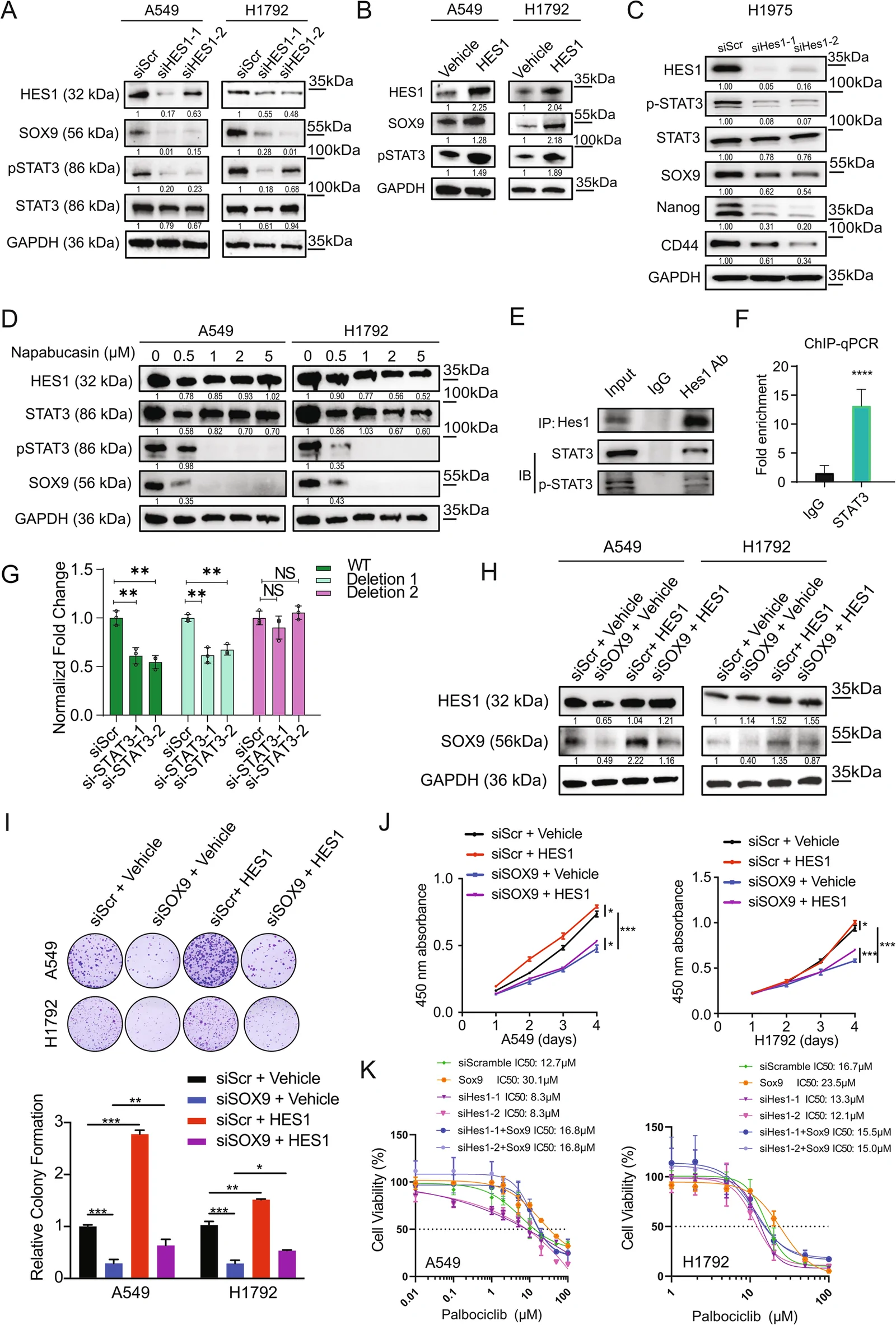
HES1 mediates SOX9 expression through STAT3 phosphorylation. **A** Western blot analysis of downstream signaling protein upon HES1 knockdown. **B** Western blot analysis after overexpression with HES1 plasmid. **C** Western blot analysis on HES1 knockdown in the EGFR-mutated cell line, H1975. **D** Western blot of A549 and H1792 cells treated with increasing concentrations of the pSTAT3 inhibitor Napabucasin (0–5 µM). **E** Co-immunoprecipitation of HES1 protein and STAT3 protein, performed with HES1 antibody (Ab). IP, Immunoprecipitation. IB, immunoblotting. **F** Chromatin immunoprecipitation (ChIP) assays performed with antibodies against IgG (control) and STAT3, and primers against the SOX9 promoter region. **G** Luciferase reporter assay with wildtype SOX9 promoter and SOX9 promoter that were deleted at the putative binding site (Deletion 2), and deleted at few base pair upstream to the putative binding site (Deletion 1). Deleted DNA sequence was shown in methods. **H** Western blot of cancer cells treated with SOX9 knockdown and rescue with HES1 overexpression plasmid. **I** Colony formation assay to assess the functional impact of HES1 rescue following SOX9 knockdown. HES1 overexpression partially restored colony formation in siSOX9-treated cells. **J** Cell proliferation assay showed HES1 overexpression rescued the proliferative capacity of siSOX9-treated cells. **K** Dose response curve towards Palbociclib of tumour cells subjected to HES1 knockdown along with rescue by SOX9 overexpression plasmid. *p < 0.05, **p < 0.01, ***p < 0.001. ****p < 0.0001. NS, not significant. siScr, siScramble. Quantification of band intensity was given for Western blot, normalized to the intensity of GAPDH

We then sought to establish the mechanistic link of HES1-mediated SOX9 overexpression. As previously reported, HES1 promotes the phosphorylation of the transcription factor STAT3, by facilitating the formation of a binding complex with the JAK2 kinase [25, 26]. We examined whether this was also responsible for SOX9 overexpression in lung adenocarcinoma. Knockdown of *HES1* resulted in a reduction in phosphorylated STAT3, and this correlated with the decrease in SOX9 protein expression (Fig. 5A). In keeping with this finding, the overexpression of HES1 led to increased phosphorylation of STAT3 (Fig. 5B). The same effect on STAT3 signaling and stemness-related markers with siHES1 transfection was also observed in another EGFR-mutated cell line, H1975 (Fig. 5C). Importantly, pharmacological inhibition of STAT3 phosphorylation by the selective inhibitor napabucasin caused the downregulation of SOX9 in a dose-dependent manner, confirming the importance of STAT3 phosphorylation toward SOX9 expression (Fig. 5D). Promoter analyses of the *SOX9* gene revealed a putative binding site for pSTAT3 that was 509 base pairs upstream to the transcription start site (Supplementary Fig. 6 C). Co-immunoprecipitation demonstrated interaction between HES1 and the phosphorylated STAT3 protein (Fig. 5E). Chromatin immunoprecipitation confirmed considerable enrichment of STAT3 at the promoter region of SOX9 (Fig. 5F). To confirm the importance of this promoter binding site, we deleted the binding site sequence from the promoter and performed the luciferase promoter assay. Compared to an intact promoter (WT) and a SOX9 promoter with sequence deleted few base pairs upstream to the predicted binding site (Deletion 1), luciferase reporter activity was not affected by siSTAT3 transfection when the SOX9 promoter binding site was deleted (Deletion 2), suggesting that the binding site was indeed crucial for STAT3 binding (Fig. 5G). Taken together, these findings confirmed that SOX9 transcription was enhanced by HES1 through phosphorylation of the transcription factor STAT3.

To confirm the association between HES1 and SOX9 on the phenotypic level, we performed rescue experiments in vitro. When SOX9 was knocked down in lung adenocarcinoma cell lines, a rescue by HES1 overexpression led to an increase in SOX9 protein expression when compared to SOX9-knockdown cells (Fig. 5H). The inhibitory effects on cell proliferation and colony formation ability were also successfully rescued by HES1 overexpression (Fig. 5I, J). Moreover, the overexpression of SOX9 could also partially abolish the effect of enhanced drug sensitivity afforded by HES1 knockdown (Fig. 5K). Collectively, these findings demonstrated that the oncogenic property of HES1 could be at least partially attributed to the downstream effect of SOX9.

### SOX9 exerts an oncogenic effect and promotes stemness in lung adenocarcinoma

**Fig. 6:**
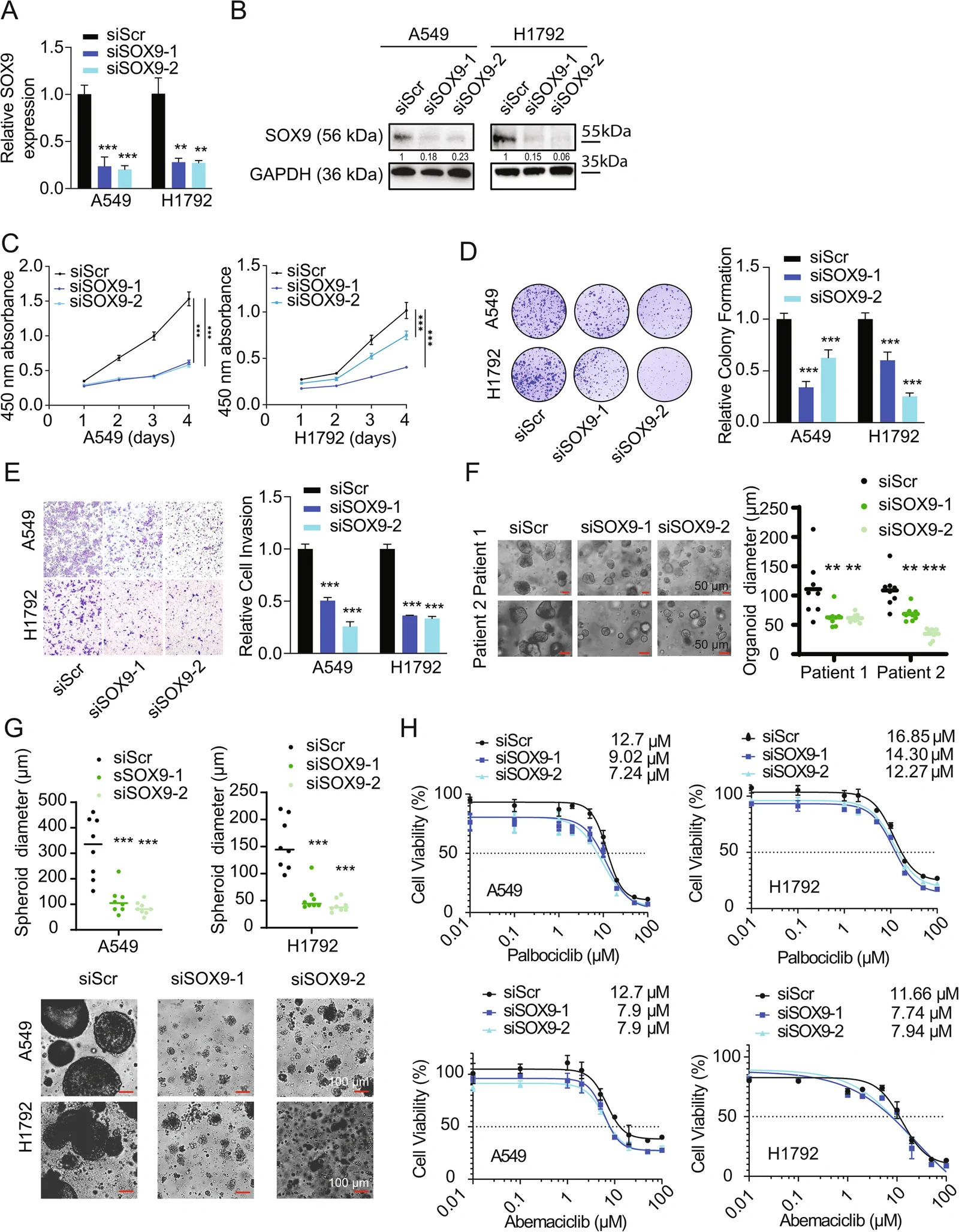
SOX9 exerts an oncogenic effect and promotes stemness in lung adenocarcinoma. **A** and **B**, SOX9 knockdown was detected by (**A**) qPCR and (**B**) Western blot. **C**, **D** and **E**, After the knockdown of SOX9 using siRNA, (**C**) cell proliferation rate, as well as (**D**) colony formation abilities and (**E**) cell invasion of the tumour cells, were significantly decreased. Student's t-test. **F**, siSOX9 transfection in the patient-derived organoids caused a reduction in the organoid size. Scale bar, 50 um. Student's t-test. **G**, In the tumour spheroid formation assay, knockdown of SOX9 resulted in a significant inhibition in spheroid formation. Student's t-test. **H**, siSOX9 transfectants showed increased sensitivity to CDK4/6 inhibitors. The IC50 value was given for each group. siScr, siScramble. **p < 0.01, ***p < 0.001. Quantification of band intensity was given for Western blot, normalized to the intensity of GAPDH

After establishing the link between HES1 and SOX9, we sought to verify the functional role of SOX9 in lung carcinogenesis. After the knockdown of SOX9 using siRNA (Fig. 6A-B), cell proliferation rate (Fig. 6C), as well as colony formation and cell invasion abilities (Fig. 6D-E) of the tumour cells, were all significantly decreased. siSOX9 transfection in the patient-derived organoids caused a reduction in the organoid size (Fig. 6F).

In the tumour spheroid formation assay, knockdown of SOX9 resulted in a significant inhibition in spheroid formation (Fig. 6G). Crucially, in siSOX9 transfectants, increased efficacy was observed towards both CDK4/6 inhibitors palbociclib and abemaciclib (Fig. 6H), and this finding phenocopied the effect of siHES1 transfection (Fig. 3J). Taken together, these data demonstrated that SOX9 exerts an oncogenic effect in lung adenocarcinoma and influences stemness, which contributes to CDK4/6 inhibitor resistance.

### CDK4/6 inhibitors can be potentiated by targeting the HES1-pSTAT3-SOX9 signalling pathway

**Fig. 7:**
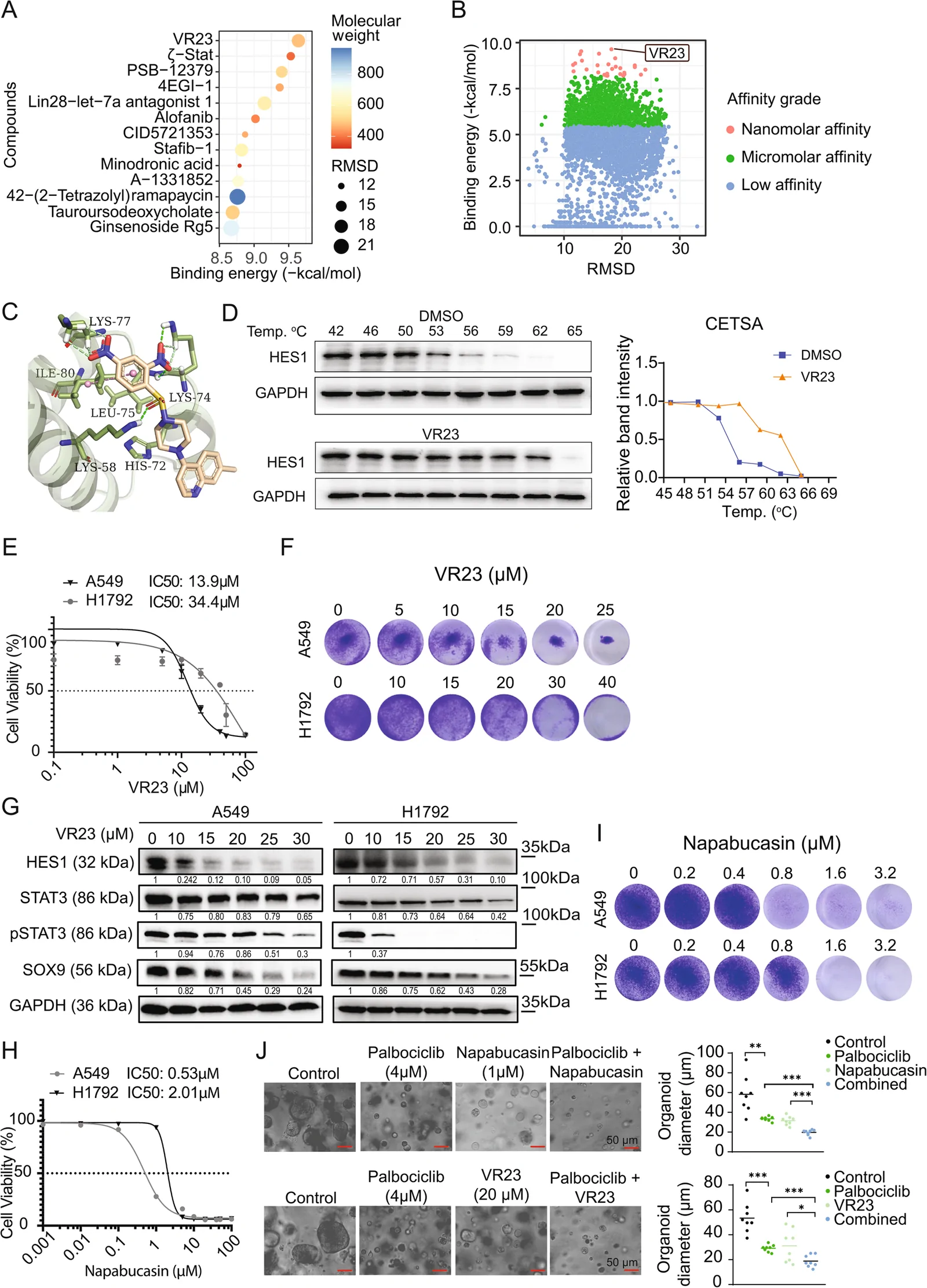
CDK4/6 inhibitors can be potentiated by targeting the HES1-pSTAT3-SOX9 signalling pathway. **A** Molecular docking analysis was performed to compare the binding energies of potential HES1 inhibitors. **B** The scatter plot showed the binding energy (in kcal/mol) and binding site RMSD values of the tested compounds, with VR23 highlighted as a high affinity binder with nanomolar affinity. **C** Molecular docking model of VR23 interacting with HES1. **D** Cellular Thermal Shift Assay showed enhanced stability of HES1 when cells were treated with VR23 at 15 µM.** E** VR23 reduced the viability of lung cancer cells. **F** The colony formation assay showed that VR23 had dose-dependent inhibitory effects on cancer cells. **G** VR23 inhibited HES1 expression, STAT3 phosphorylation, and downstream SOX9 expression in a dose-dependent manner. **H** and **I** Napabucasin inhibited the (**H**) cell proliferation and (**I**) colony formation of the adenocarcinoma cell lines in micromolar concentration. **J** Combination treatment of palbociclib with VR23 or napabucasin in patient-derived organoids resulted in a reduction in organoid size. Student's t-test. Quantification of band intensity was given for Western blot, normalized to the intensity of GAPDH

To explore the therapeutic vulnerability conferred by the HES1 signaling pathway, we performed a high-content small molecule screening from the drug library to identify potential HES1 inhibitors. With molecular docking analyses, a number of small molecules were found to exhibit potent binding with HES1 (Fig. 7A), and VR23 emerged as a compound that possessed nanomolar affinity to HES1 (Fig. 7B). It was modelled that the molecule bound to its target by hydrogen bond and intermolecular van der Waals force to several amino acid residues (Fig. 7C, Supplementary Fig. 7 A). Cellular thermal shift assay (CETSA) revealed that VR23 treatment remarkably increased the stability of HES1 protein, indicating the direct binding between HES1 and VR23 (Fig. 7D). Similar results were observed with Drug affinity responsive target stability (DARTS) assay, where an increased VR23 concentration stabilized the HES1 protein and prevented it from pronase digestion (Supplementary Fig. 7B). In vitro, VR23 showed an inhibitory effect on the cancer cell lines (Fig. 7E), and reduced their colony formation abilities in a dose-dependent manner (Fig. 7F). Mechanistically, the administration of VR23 resulted in a decrease in protein expression of HES1, as well as a reduction in STAT3 phosphorylation and SOX9 protein expression (Fig. 7G). The mRNA expression level of *HES1* was unaffected by VR23 administration (Supplementary Fig. 7 C), and the protein expression of proteotoxic markers, such as the heat shock protein family members, were also unaffected (Supplementary Fig. 7D), suggesting that the inhibitory effect of VR23 was not bought about by transcriptional suppression or by a global proteotoxic effect. These data confirmed the inhibition of STAT3-mediated stemness pathway by VR23 through targeting HES1.

**Fig. 8:**
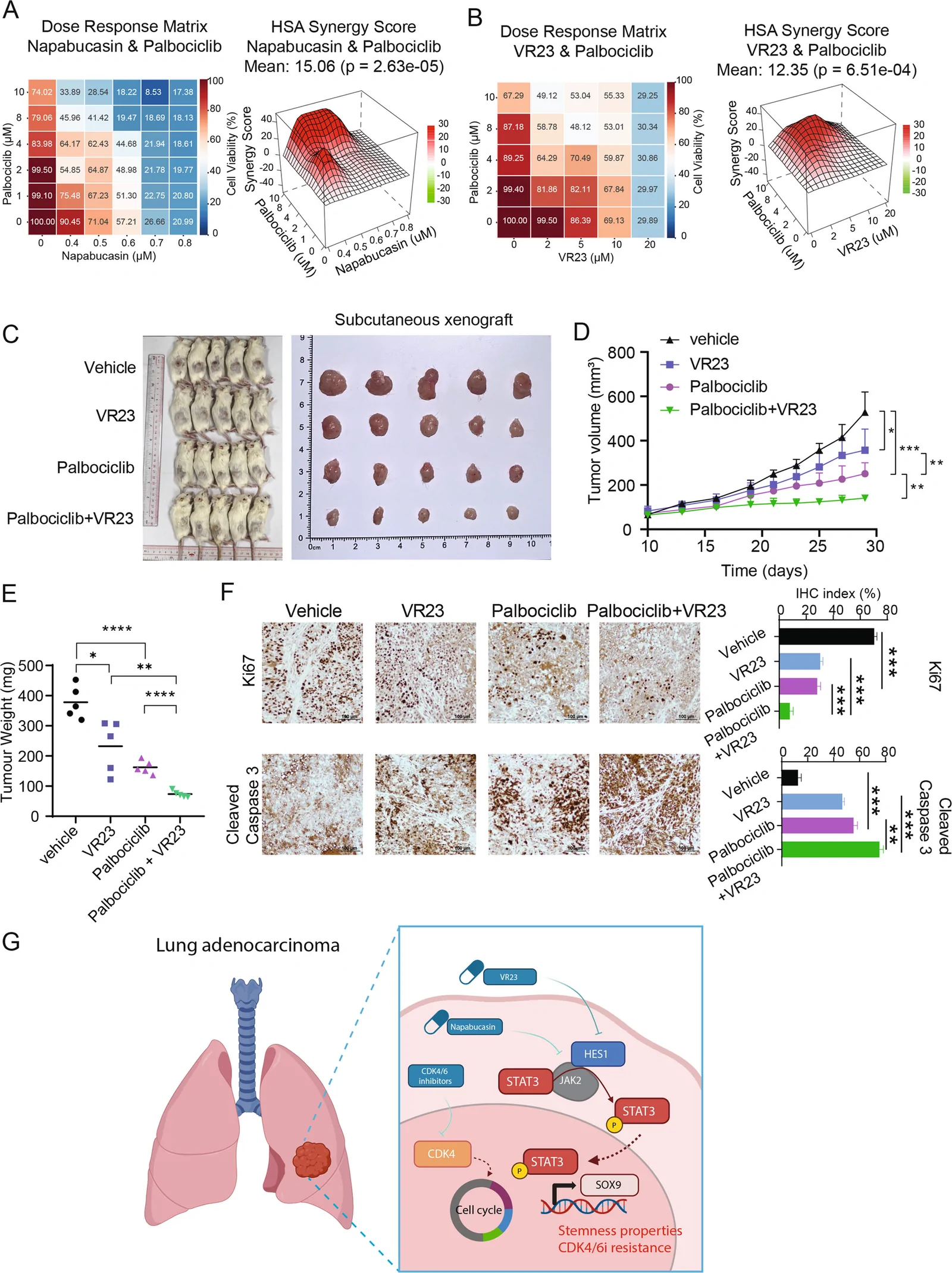
Combination therapy in vivo demonstrates synergistic effect with CDK4/6 inhibitor and HES1 inhibitor. **A** and **B** Dose–response analyses of the drug combination of (**A**) napabucasin plus palbociclib, and (**B**) of VR23 and palbociclib confirmed synergistic effect, with the red peak of the three-dimensional plot showing a high HSA synergy score. **C** Subcutaneous xenografts in treatment groups with VR23 alone, Palbociclib alone or both drugs combined. *n =* 5 mice per group. **D** Tumour volume of the subcutaneous xenograft along the course of treatment. *n =* 5 mice per group. **E** Tumour weight at the time of sacrifice and harvest of subcutaneous xenograft. Student's t-test. *n =* 5 mice per group. **F** Representative photomicrograph of Ki67 and cleaved caspase 3 immunohistochemistry (IHC) and quantification with IHC index. **G** Schematic diagram of the key findings in this paper, highlighting the role of HES1-pSTAT3-SOX9 in mediating cancer stemness and CDK4/6 inhibitor resistance, and the therapeutic vulnerabilities offered by HES1 and STAT3 phosphorylation targeting

With this observation, we reasoned that inhibiting STAT3 phosphorylation should also be a viable therapeutic approach. Indeed, the selective inhibitor of STAT3 phosphorylation, napabucasin, could phenocopy the downstream inhibition of SOX9 by VR23 on Western blot (Fig. 5D) in a dose-dependent manner. Napabucasin could also inhibit the growth and colony formation of the adenocarcinoma cell lines in micromolar concentration (Fig. 7H, I). To test the use of combination therapy with CDK4/6 inhibitor and inhibitors of the HES1-pSTAT3-SOX9 signalling pathway, we treated patient-derived organoids with palbociclib alone and in combination with napabucasin or VR23. The inhibitory effect on organoid growth was more prominent with the drug combination palbociclib + VR23 and palbociclib + napabucasin, compared to either drug alone (Fig. 7J), suggestive of a synergistic effect. Dose–response analyses of the drug combination confirmed such synergy, with the red peak of the three-dimensional plot showing a high HSA synergy score for both drug combinations (Fig. 8A-B).

To confirm the therapeutic effect of the drug combination in-vivo, we administered Palbociclib and VR23 to tumour-bearing mice over a course of 30 days (Fig. 8C). At the end-point, significantly lower tumour volume and tumour weight were observed in mice which were given the Palbociclib and VR23 drug combination (Fig. 8D, E), when compared to groups which were given the vehicle or either drug alone. Immunohistochemically, the tumour cells in the drug combination group exhibited significantly lower Ki67 cell proliferation index and an elevated cleaved caspase 3 level, an apoptosis marker (Fig. 8F). The in-vivo model demonstrated that using VR23 with Palbociclib may afford a synergistic effect and is of clinical translational potential.

In conclusion, our results showed that the resistance of CDK4/6 inhibitors in lung adenocarcinoma was conferred by the HES1-mediated SOX9 overexpression, which in turn led to enhanced cancer stemness properties and contributed to drug resistance. This resistance toward CDK4/6 inhibitors could be overcome by targeting individual signalling molecules within this pathway, such as by HES1 inhibitor, or STAT3 phosphorylation inhibitor. When combined with CDK4/6 inhibitors, the downregulation of SOX9 and cancer stemness properties by VR23 or napabucasin could result in a synergistic anti-tumour effect (Fig. 8G).

## Discussion

CDK4/6 inhibitors have emerged as an effective targeted therapy in breast cancer, but its effectiveness in lung cancer remains to be further clarified. As a monotherapy, the CDK4/6 inhibitor Palbociclib appeared to have only a modest tumour response in a cohort of 29 patients [9]. Because CDK4 alterations are relatively common in lung cancer compared to other cancers, the inhibition of this pathway is a therapeutic avenue worth deeper investigation. Importantly, the suboptimal efficacy as demonstrated by clinical trials suggest there is a need to develop new therapeutic strategies with CDK4/6 inhibitors, such as a combination approach or the targeting of its resistance mechanism.

While a number of ongoing clinical trials are assessing the outcomes of CDK4/6 inhibitors combined with tyrosine kinase inhibitors [27] or ALK inhibitors [28], preclinical investigations are also shedding light to the underlying therapeutic mechanisms of CDK4/6 inhibitors. Early studies demonstrated that the synergistic inhibition of the ERK signalling with MEK inhibitors potentiates CDK4/6 inhibitors in colorectal cancer [29, 30]. More recently, CDK4/6 inhibitors were found to overcome LKB1 mutation-mediated immunotherapy resistance in KRAS-driven lung adenocarcinoma [31]. Meanwhile, major resistant mechanisms were thought to be related to overexpression of other cell cycle molecules such as CCNE1, activation of the kinases CHEK1 or AURKA [21, 32], or alterations in the Hippo pathway [33].

Whether the stemness properties in lung adenocarcinoma is implicated in CDK4/6 inhibitor resistance was largely unexplored. HES1 is a transcription factor implicated in the Notch signaling pathway, which is involved in cell fate decisions and stem cell maintenance, and is physiologically important in embryonic development, particularly in the nervous system [15]. HES1 was traditionally thought to be a transcriptional repressor by binding to its basic helix-loop-helix domain or recruitment of histone deacetylases [17], but in other context also activates RUNX2 expression and STAT3 phosporylation [25], exerting a generally oncogenic effect [34]. Because we observed HES1 upregulation in lung adenocarcinoma and CDK4/6 inhibitor resistant cell lines, we wanted to dissect its downstream signalling pathway and examine its potential as a therapeutic target.

We identified CDK4/6 amplification and overexpression is a common event in lung adenocarcinoma, and found that CDK4/6 inhibitors could exert an anti-tumour effect in patient-derived organoids. Further, we characterized the oncogenic effect of HES1 and observed that its knockdown enhanced the efficacy of CDK4/6 inhibitors in-vitro. Crucially, HES1-depleted spheroids were also more responsive to treatment with palbociclib, alluding that cancer stemness may play a role in influencing CDK4/6 inhibitor responses.

Consistent with this notion, geneset enrichment analysis showed that a stem cell differentiation and maintenance was associated with HES1 expression, and SOX9 was ranked as one of the top downstream candidates. Our results were supported by clinical data, as SOX9 expression positively correlated with that of HES1 in published database. We confirmed that the stemness properties brought about by SOX9 was activated by HES1-mediated STAT3 phosphorylation. Macromolecular modelling analysis identified VR23 as a small molecule that antagonized HES1 and synergized with CDK4/6 inhibitor to exert an anti-tumour effect in patient-derived organoids and in mice. Importantly, inhibition of STAT3 phosphorylation by napabucasin also phenocopied this anti-tumour effect, providing consistent evidence that the HES1-pSTAT3-SOX9 mediated stemness was an important target to mitigate CDK4/6 inhibitor resistance. With the development of novel selective CDK4 inhibitor, this finding is expected to be useful in considering combination therapy in treating lung cancer patients.

A limitation in this study was that there were no data from patients who were treated with CDK4/6 inhibitors. This was because CDK4/6 inhibitors have yet to be standard therapy in lung cancer patients, and patient materials for detailed mechanistic study of resistance mechanisms were lacking. Although we identified HES1 overexpression in cell lines and organoids with reduced drug responsiveness, further validation with samples from lung cancer patients who were treated by CDK4/6 inhibitors will be required to assess the role of HES1 as a predictive biomarker. The observed micromolar IC50 in cell lines, while comparable to the published database [35], may exceed clinically achievable plasma concentrations. This may be due to the potential intrinsic resistance towards CDK4/6 inhibitors in these cell lines [8], and the high proliferation rate of cell lines that accounted for a higher concentration needed for inhibition [36]. Although off-target effect of CDK4/6 inhibitors could not be completely excluded at this concentration, these data gave insights to further preclinical studies and were of translational relevance.

Taken together, our study dissected a novel HES1-mediated cancer stemness mechanism that has important translational relevance to using CDK4/6 inhibitors in lung adenocarcinoma patients. We also identified inhibition of the cancer stemness property with small molecules could potentiate the effect of this therapeutic agent. Given the prevalence of CDK4 alterations in lung cancer, our findings demonstrated that CDK4/6 inhibitors could be a useful therapeutic modality for lung adenocarcinoma patients while a deeper mechanistic understanding would be crucial to enhance the treatment outcome. This serves as a promising target for further drug development and clinical investigations.

## Conclusion

CDK4/6 inhibitors are targeted therapy with high potential especially for lung cancer with no classical druggable target. Nevertheless, to overcome the low response rate of patients towards these agents, a combination therapy approach is necessary. Cancer stemness in lung adenocarcinoma, as mediated by the Notch-Hes1 signaling pathway, is important in mediating resistance towards CDK4/6 inhibitors. This study demonstrated that each signaling molecule in the HES1-STAT3-SOX9 axis can be targeted to achieve a synergistic effect with CDK4/6 inhibitors. Specifically, a novel HES1 inhibitor, VR23, as well as the STAT3 phosphorylation inhibitor, napabucasin, can both exert synergy with palbociclib. In particular, the combination of napabucasin and palbociclib, both being established drugs with FDA approval, are attractive candidates for clinical trials. Targeting the Notch-Hes1 signaling represents a treatment strategy that can potentiate the efficacy of CDK4/6 inhibitor in lung adenocarcinoma patients.

## Supplementary Information

Supplementary Material 1.

## Data Availability

All cell lines, vectors and other stable reagents generated in this study are available from the corresponding author. The datasets used or analyzed are available on reasonable request.

## Abbreviations

HES1: Hairy and Enhancer of Split 1
CDK4: Cyclin-dependent kinase 4
CDK6: Cyclin-dependent kinase 6
SOX9: SRY-box transcription factor 9
STAT3: Signal transducer and activator of transcription 3
pSTAT3: Phosphorylated STAT3
Rb: Retinoblastoma protein
EGFR: Epidermal growth factor receptor
KRAS: Kirsten rat sarcoma viral oncogene
ALK: Anaplastic lymphoma kinase
PARP: Poly (ADP-ribose) polymerase
MEK: Mitogen-activated protein kinase kinase
ERK: Extracellular signal-regulated kinase
NANOG: Nanog homeobox
ALDH1: Aldehyde dehydrogenase 1
CD44: Cluster of differentiation 44
CD133: Cluster of differentiation 133
ABCG2: ATP-binding cassette sub-family G member 2
GAPDH: Glyceraldehyde 3-phosphate dehydrogenase
NSCLC: Non-small cell lung cancer
IHC: Immunohistochemistry
FISH: Fluorescence in situ hybridization
ChIP: Chromatin immunoprecipitation
qPCR: Quantitative polymerase chain reaction
SDS-PAGE: Sodium dodecyl sulfate polyacrylamide gel electrophoresis
GSEA: Gene set enrichment analysis
TCGA: The Cancer Genome Atlas
GEO: Gene Expression Omnibus
ATCC: American Type Culture Collection
GDSC: Genomics of Drug Sensitivity in Cancer
