# Supplementary material for: HES1 inhibition overcomes CDK4/6 inhibitor resistance by targeting cancer stemness in lung adenocarcinoma
Source: J Exp Clin Cancer Res. 2026 Jun 27;45:195. doi: 10.1186/s13046-026-03771-x (PMC13576211; doi:10.1186/s13046-026-03771-x)
Supplement: Supplementary file 1 [file 13046_2026_3771_MOESM1_ESM.pdf]

Supplementary information

HES1 Inhibition Overcomes CDK4/6 Inhibitor Resistance by Targeting Cancer Stemness in Lung Adenocarcinoma

Alvin Ho-Kwan Cheung, Kit Yee Wong, Gordon Yuan-Ho Li, et al.

Supplementary Figure 1

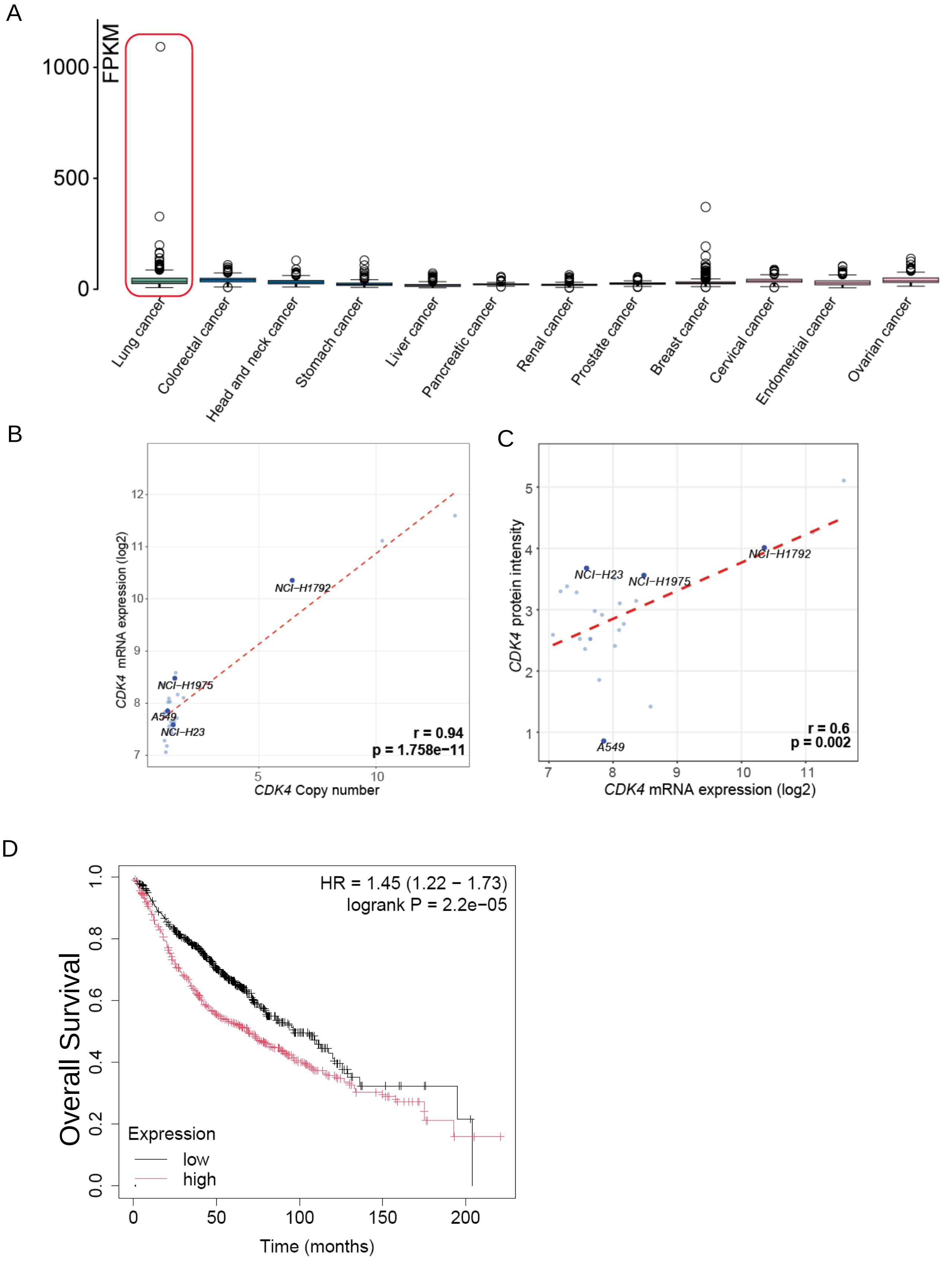

Supplementary Figure 1. *CDK4* is a crucial oncogenic event in lung adenocarcinoma with prognostic impact. (A) Pan-cancer profile of *CDK4* RNA expression level from the Human Protein Atlas. (B-C) The correlation of (B) CDK4 copy number and mRNA expression and (C) the mRNA expression and protein intensity according to DepMap database. Only the names of cell lines used in this study were labeled. Only cell lines with data in all three of the copy number, mRNA expression and protein intensity were included in the analysis. Pearson correlation. (D) Survival curve modified from the Kaplan-meier plotter database which includes TCGA and GEO lung adenocarcinoma dataset for analyses.

Supplementary Figure 2

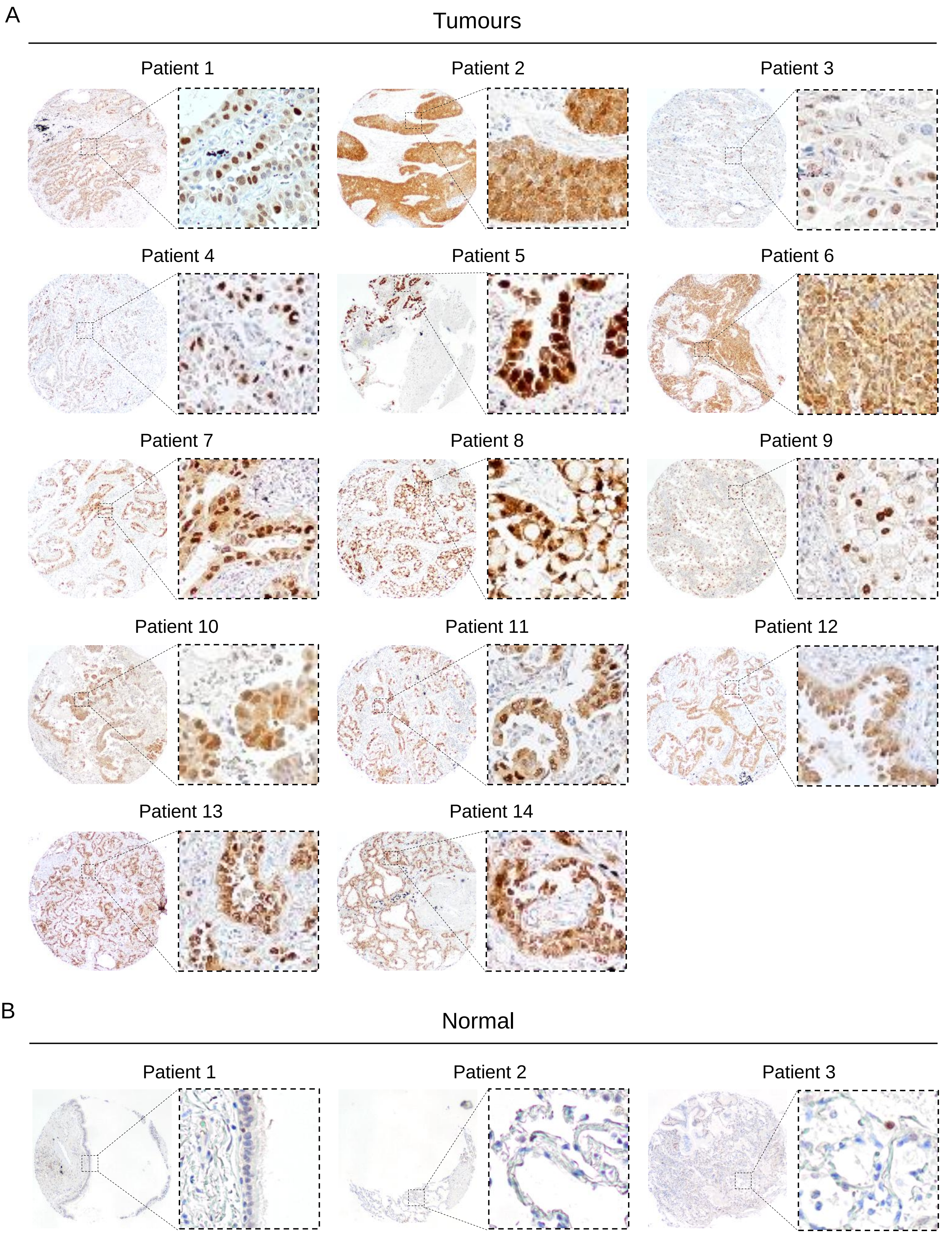

Supplementary Figure 2. *CDK4* expression in tissue microarray. (A) CDK4 protein expression was observed in the tumour cells. (B) Immunostaining of CDK4 in tissue microarray with normal anatomical components.

Supplementary Figure 3

A

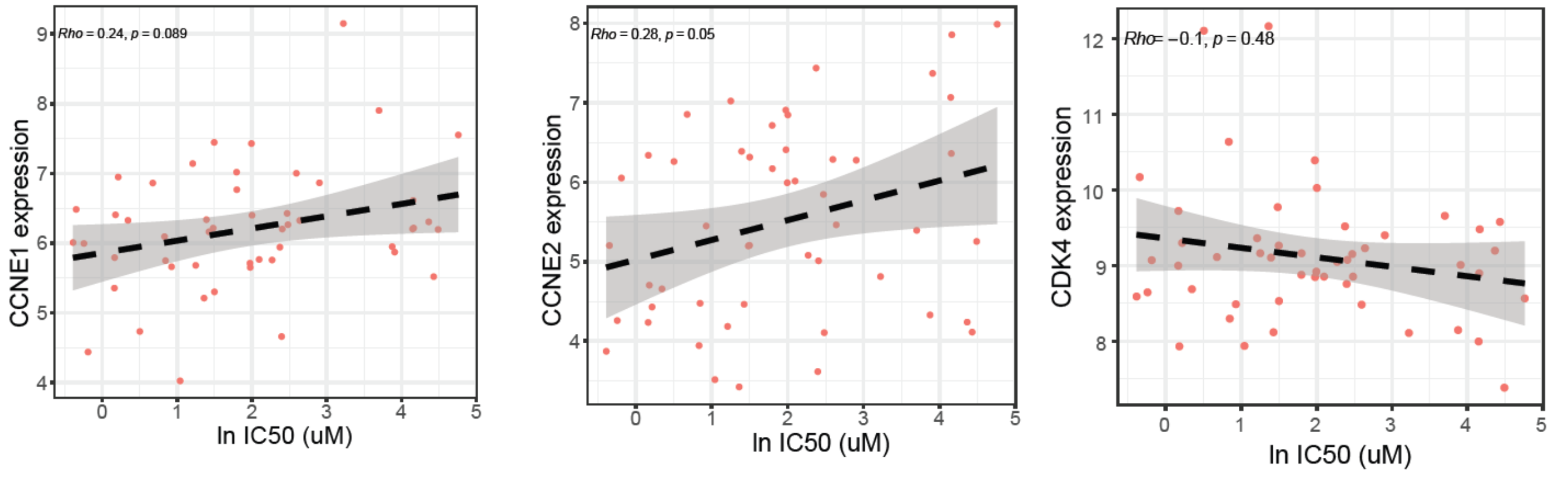

B

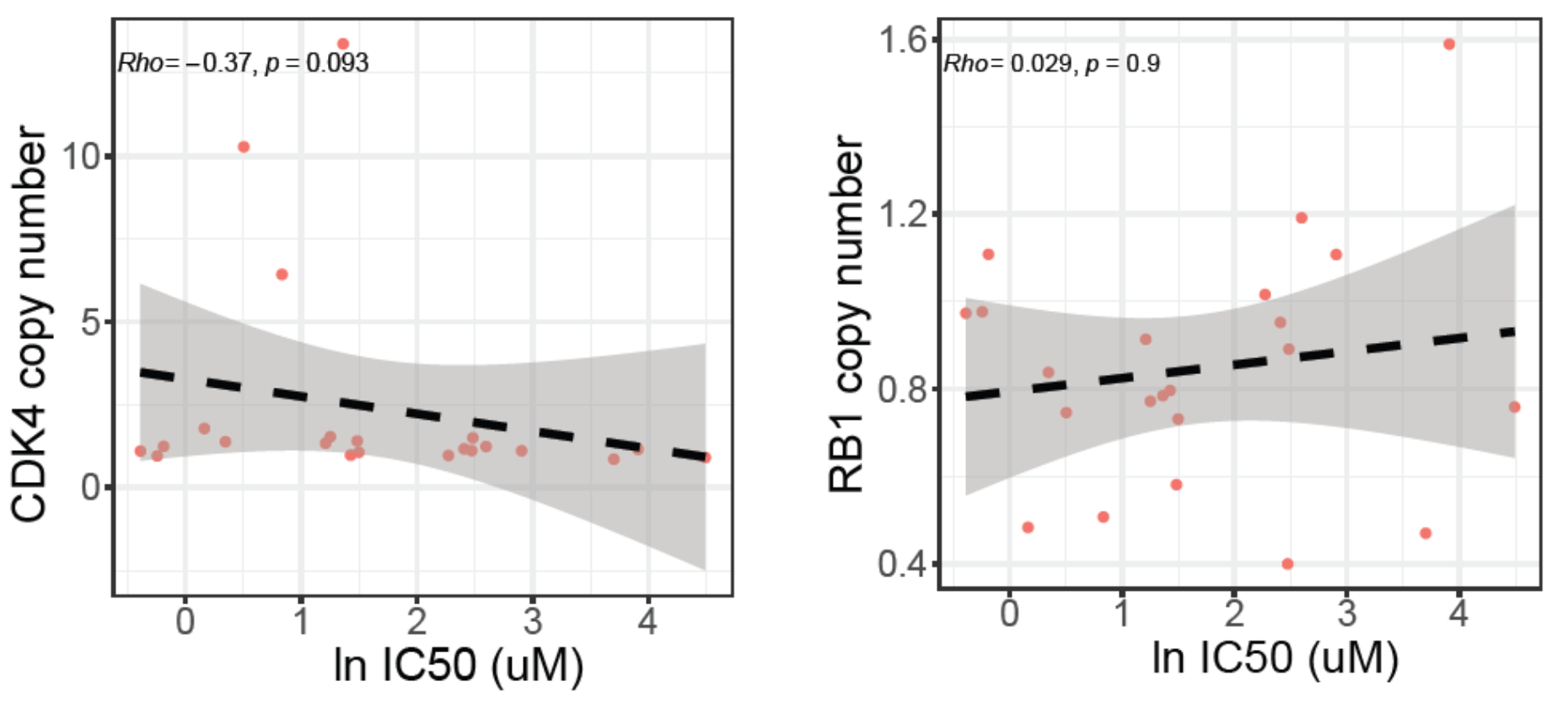

Supplementary figure 3. Correlation of protein expression and copy number of genes with drug sensitivity. (A) Correlation between CCNE1, CCNE2, CDK4 expression and drug sensitivity. (B) Correlation between the copy number of CDK4 and RB1 and drug sensitivity.

Supplementary Figure 4

A

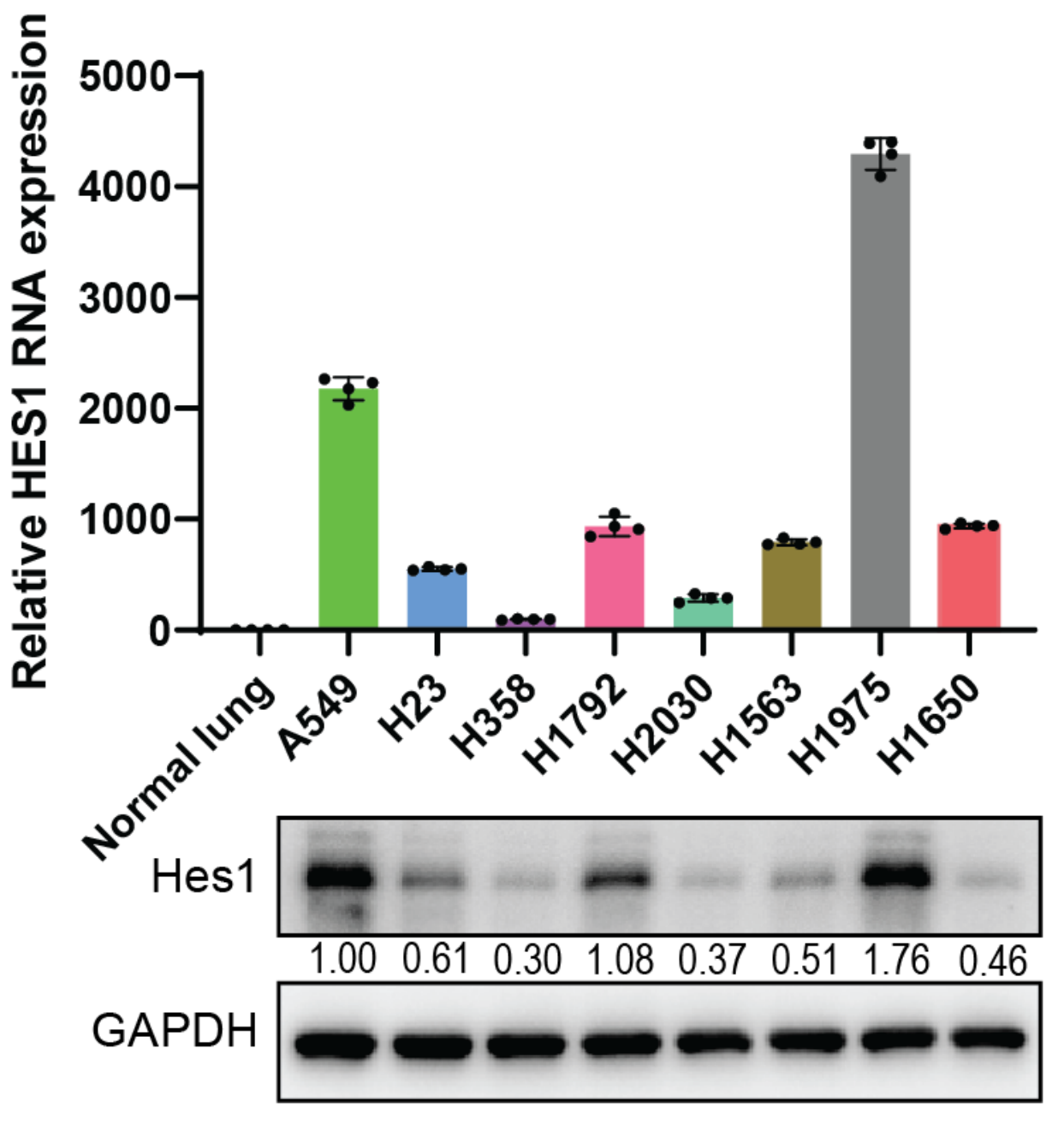

B

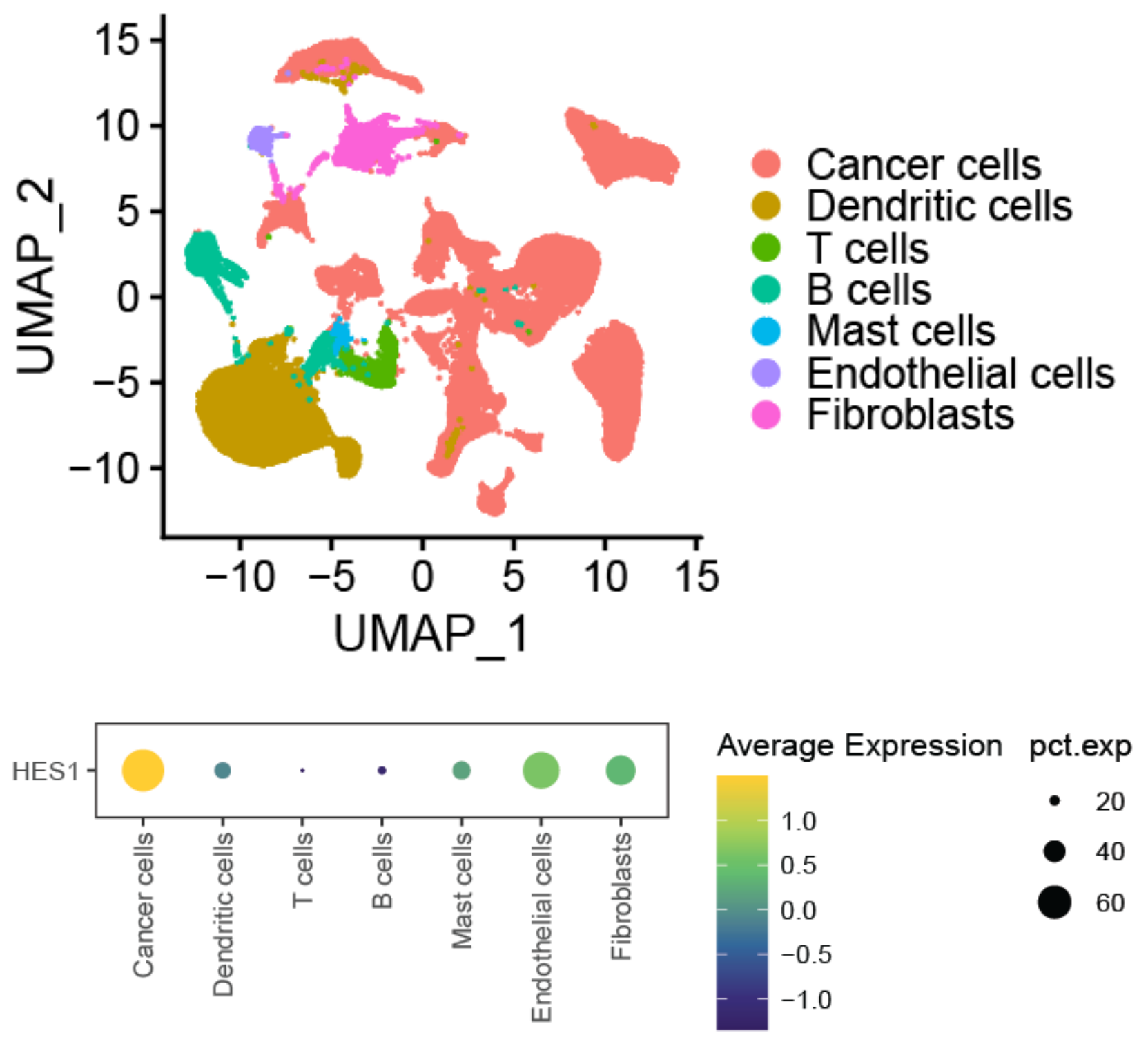

Supplementary figure 4. HES1 expression across clusters in lung cancer (A) HES1 mRNA expression and protein expression in lung cancer cell lines. (B) (Top) UMAP plot showed the clustering of different cell types, including cancer cells, dendritic cells, T cells, B cells, mast cells, endothelial cells, and fibroblasts, with each color representing a distinct cell type. (Bottom) Dot plot displayed HES1 expression levels across different cell types.

Supplementary Figure 5

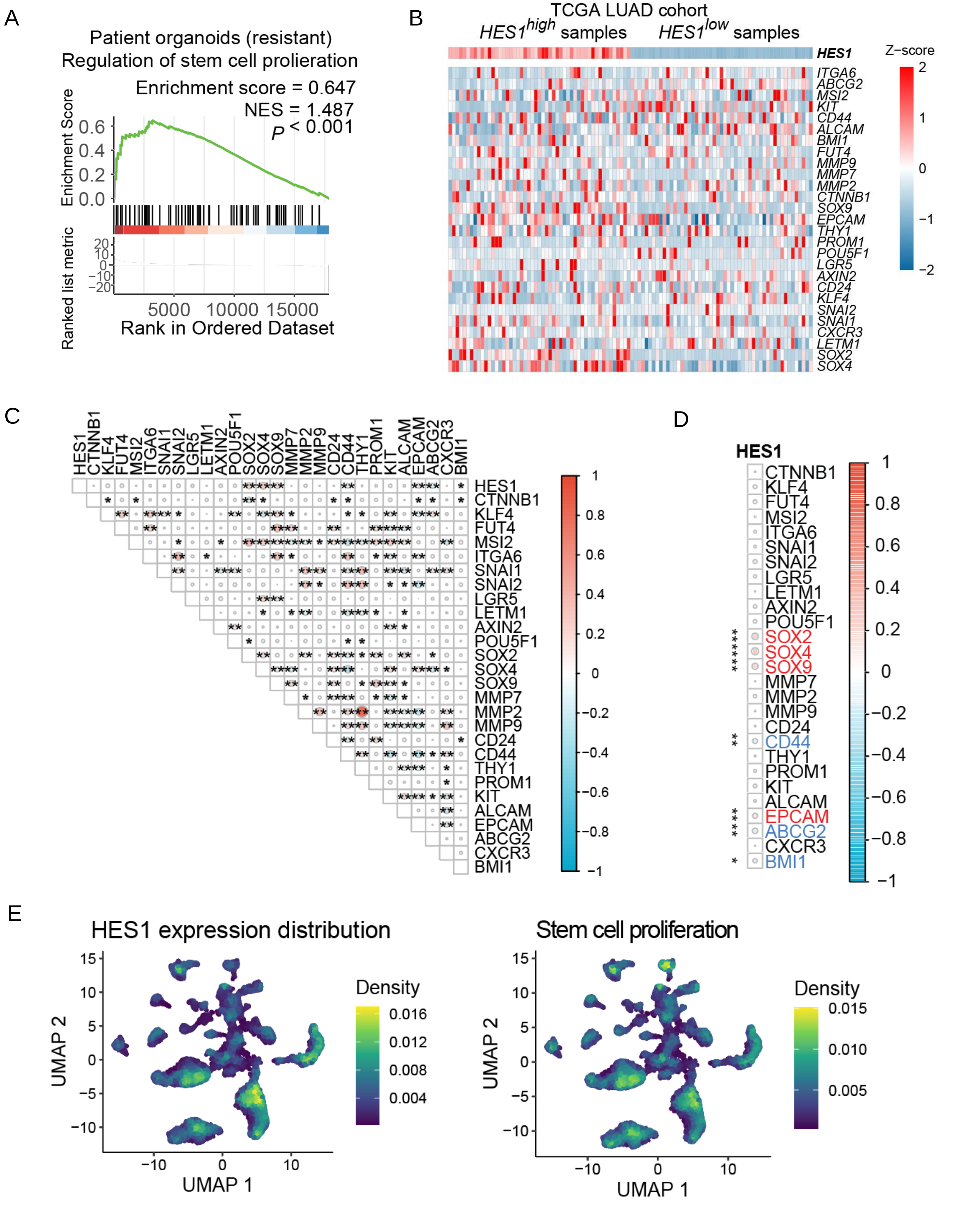

Supplementary figure 5. HES1 expression and its correlation with stemness in LUAD. (A) Gene set enrichment analysis (GSEA) of patient derived organoids exhibiting lower drug sensitivity (Patients R1-R3) compared to those exhibiting higher drug sensitivity (Patients S1-S3). (B) Heatmap of key gene expression in TCGA LUAD samples, grouped by $HES1^{high}$ and $HES1^{low}$. (C) Correlation matrix showed relationships between HES1 and stemness-related genes. Colors represent Pearson correlation coefficients; asterisks indicated significance (*p < 0.05, **p < 0.01, ***p < 0.001). (D) Candidate genes that were enriched in lung cancer with high HES1 expression in the TCGA cohort. (E) UMAP plots displayed HES1 expression distribution (left) and its association with stem cell proliferation (right) in cancer cells.

Supplementary figure 6

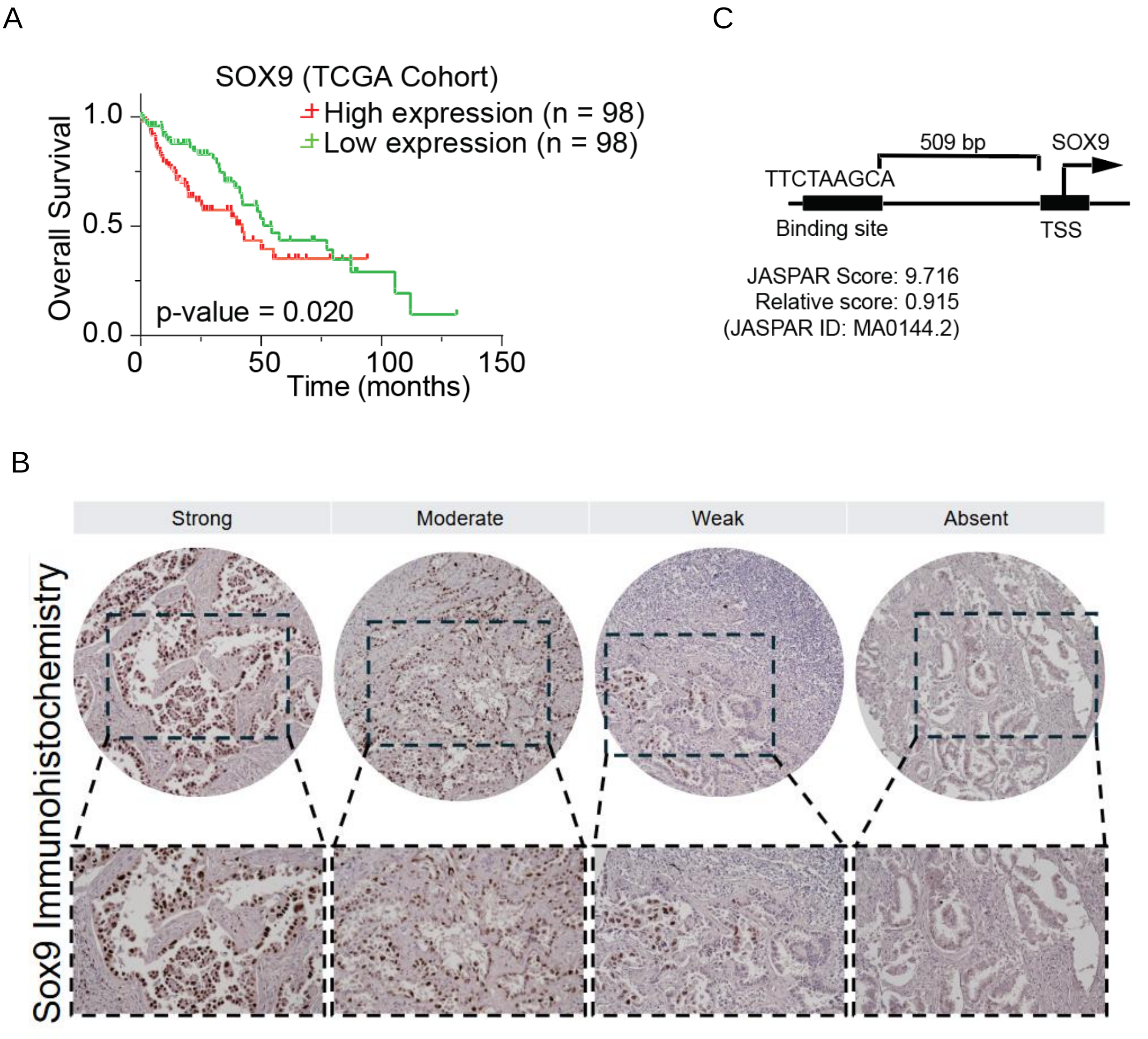

Supplementary figure 6. SOX9 expression in lung cancer tissues.
A. Kaplan-Meier survival analysis of the TCGA cohort in patients stratified by SOX9 expression, with high expression defined as the highest 20% expression and low expression as the lowest 20% expression. B. Representative images showed strong, moderate, weak, and absent Sox9 expression on immunohistochemistry. C. Predicted binding site of SOX9 promoter in JASPAR database

Supplementary Figure 7

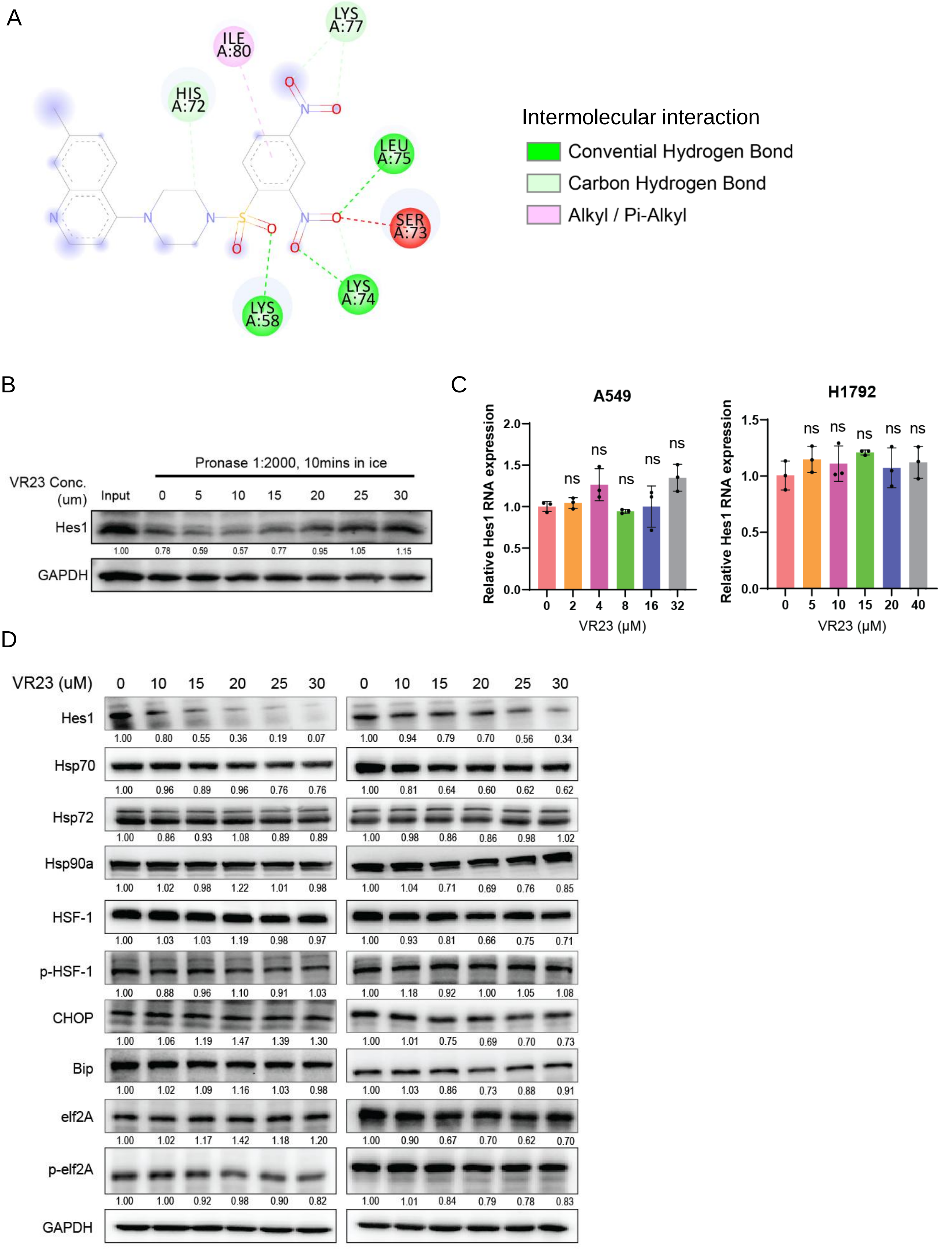

Supplementary figure 7. Molecular docking analysis of the interaction between VR23 and HES1. (A) 2D interaction diagram showed the binding of the VR23 to its target protein HES1. (B) Pronase digestion showed retained HES1 with increased concentration of VR23. (C) HES1 transcription appeared unaffected by VR23 treatment. NS, not significant. (D) Western blot of markers relating to proteotoxic stress on VR23 treatment.
